# Resolved HBV Infection Is Not Associated With Liver‐Related Outcomes in Survival Analysis of Caucasians After HCV Cure

**DOI:** 10.1111/liv.70620

**Published:** 2026-04-30

**Authors:** Laura Muana Wilhelm, Albrecht Stoehr, Peter Buggisch, Christine John, Ralph Link, Hartwig Klinker, Uta Merle, Markus Cornberg, Christoph Sarrazin, Thomas Berg, Heiner Wedemeyer

**Affiliations:** ^1^ Department of Gastroenterology, Hepatology, Infectious Diseases and Endocrinology Hannover Medical School Hannover Germany; ^2^ Ifi‐Institute for Interdisciplinary Medicine Hamburg Germany; ^3^ Center of Gastroenterology Berlin Germany; ^4^ MVZ‐Offenburg GmbH/St. Josefs‐Klinik Offenburg Germany; ^5^ Division of Infectious Diseases, Department of Internal Medicine II University of Würzburg Medical Center Würzburg Germany; ^6^ Department of Gastroenterology, Hepatology and Infectious Diseases Heidelberg University Hospital Heidelberg Germany; ^7^ Centre for Individualised Infection Medicine (CiiM) Hannover Germany; ^8^ St. Josefs‐Hospital Wiesbaden Germany; ^9^ Goethe University Hospital Frankfurt Germany; ^10^ Division of Hepatology, Department of Medicine II Leipzig University Medical Center Leipzig Germany; ^11^ Leberstiftungs‐GmbH Deutschland Hannover Germany; ^12^ German Center for Infection Research (DZIF) Braunschweig Germany; ^13^ Hannover Medical School Resolving Infection Susceptibility (RESIST) Hannover Germany

**Keywords:** anti‐HBc positivity, hepatitis C virus cure, hepatocellular carcinoma, liver‐related events, resolved hepatitis B virus infection

## Abstract

**Background:**

Previous exposure to hepatitis B virus (HBV) may influence the risk of developing hepatocellular carcinoma (HCC) and other liver‐related events (LRE), in particular in patients after HCV cure. Previous studies were not conclusive and there are only few large studies on this topic from Europe.

**Methods:**

We analysed clinical endpoints (≥ 3‐point increase in MELD score, oesophageal variceal bleeding, ascites, encephalopathy, liver transplantation, death, with/without HCC; HCC alone) in patients cured from HCV. Data were obtained from the German Hepatitis C Registry. Patients after organ transplantation, a history of HCC, HIV co‐infection, or HBsAg positivity were excluded. A subanalysis was conducted in patients with cirrhosis. Statistical analyses included logistic regression to identify predictors of clinical endpoints and Kaplan–Meier curves to analyse the influence of HBV serological markers.

**Results:**

A cohort of 6198 patients fulfilled inclusion criteria, the median time of follow‐up was 2.5 years (range 0.04–8.01). Serological evidence of previous HBV exposure was present in 1889 patients (anti‐HBc positive). In patients with cirrhosis, univariate analyses identified anti‐HBc positivity (odds ratio [OR], 1.48), cirrhosis (OR, 4.89), features of portal hypertension (ascites (OR, 5.66), oesophageal varices (OR, 4.88)), diabetes (OR, 3.23), and malignancies (OR, 10.34) as risk factors for composite LRE. In multivariable analysis, anti‐HBc positivity (OR, 1.53) and cirrhosis (OR, 4.63) remained independent risk factors for the composite endpoints, whereas anti‐HBc positivity was not associated with HCC or Kaplan–Meier survival analyses.

**Conclusions:**

Resolved HBV infection was not associated with the development of HCC or survival in Caucasians after HCV cure. Although anti‐HBc positivity was linked to composite outcomes, its clinical relevance appears limited.

**Trial Registration:**

The registry was registered at the German Clinical Trials Register (DRKS; IDDRKS00009717).

## Lay Summary

Previous infection with hepatitis B virus (HBV), which often goes undetected, has been discussed as a potential risk factor for liver‐related complications after hepatitis C (HCV) cure. In this large study of Caucasian patients who successfully cleared HCV, resolved HBV infection was not associated with individual liver‐related events (HCC) or reduced survival. Although markers of past HBV exposure were linked to combined liver outcomes, their overall clinical relevance appears limited.

**Abbreviations**
APRI: aspartate aminotransferase/platelet ratio index
AST: aspartate aminotransferase
BL: baseline
BMI: body mass index
cccDNA: covalently closed circular DNA
CI: confidence interval
DAA: direct‐acting antiviral
DCV: daclatasvir
DHC‐R: German Hepatitis C‐Registry
DRKS: German Clinical Trials Register
DSV: dasabuvir
DZIF: German Center for Infection Research
EBR: elbasvir
EoT: end of treatment
FIB4: fibrosis‐4
FU: follow‐up
GLE: glecaprevir
GZR: grazoprevir
HBc: Hepatitis B core antigen
HBsAg: hepatitis B surface antigen
HBV: hepatitis B virus
HCC: hepatocellular carcinoma
HCV: hepatitis C virus
HIV: human immunodeficiency virus
IFN: interferon
LDV: ledipasvir
LRE: liver‐related events
MELD: model for end‐stage liver disease
OBI: occult hepatitis B
OBV: ombitasvir
OR: odds ratio
PIB: pibrentasvir
PTV/r: paritaprevir/ritonavir
RBV: ribavirin
SOF: sofosbuvir
SVR: sustained virologic response
Tx: transplantation
VEL: velpatasvir
WHV: woodchuck hepatitis virus

## 1 Introduction

The natural course of hepatitis B virus (HBV) infection varies widely, ranging from acute, self‐limiting infection to chronic liver disease and hepatocellular carcinoma (HCC). Resolved HBV infection, while indicative of successful viral clearance, does not always equate to the complete eradication of HBV from the body. A subset of individuals may continue to harbour covalently closed circular DNA (cccDNA) within hepatocytes [1, 2, 3]. The persistence of replication‐competent HBV genomes is known as occult hepatitis B infection (OBI), which is classically defined by the absence of detectable HBsAg in serum in the presence of detectable HBV DNA in liver tissue and, in some cases, at low levels in peripheral blood, usually in individuals who are anti‐HBc positive [1]. In the present study, HBV DNA data were not available in the majority of patients. Consequently, we use the term “resolved HBV infection” throughout this manuscript, in accordance with established serological definitions. Resolved HBV infection poses significant clinical implications, particularly in the context of immunosuppression, which can trigger reactivation of the virus and lead to acute hepatitis or fulminant liver failure [1]. Additionally, it complicates organ transplantation protocols due to the risk of HBV transmission from donors with resolved infection or reactivation of an occult pre‐transplant infection [4]. The impact of resolved HBV infection on the development of HCC and liver‐related events (LRE) remains a contentious issue in the medical community. Resolved infection seems to retain oncogenic potential typically associated with HBsAg positive HBV infections. Its exact role as a risk factor for HCC and other LRE remains unclear [1]. Numerous studies have demonstrated a significant association between resolved HBV infection and HCV‐related HCC [5, 6, 7] and non‐HCV‐related HCC [8]. Others have reported no such association [9]. However, there are only a few studies on this clinically important topic from Europe [6, 10].

We aimed to investigate the impact of previous transient HBV infection on the development of HCC and other LRE in a large prospective cohort of well‐characterized HBsAg negative patients with cured HCV infection.

## 2 Methods

### 2.1 Study Design and Study Population

For the analysis of clinical endpoints after achievement of SVR in the presented real‐world study, data were obtained from the German Hepatitis C‐Registry (DHC‐R), a national real‐world cohort of approximately 18 900 HCV infected patients to date (German Clinical Trials Register [Deutsches Register Klinischer Studien, DRKS] ID DRKS00009717). Patients were required to provide written informed consent to be enrolled in the registry. Baseline (BL) was defined as the time point of SVR and SVR was defined as undetectable or a ≤ 25 IU/mL viral load in quantitative PCR 12 to 24 weeks (FU12/24) after end of treatment (EoT). The median time of follow‐up was 2.5 years (range 0.04–8.01), the mean follow‐up duration was 3.0 ± 1.9 years. Collected baseline data comprised sociodemographic information (age and sex), lifestyle factors (alcohol and coffee consumption), HBV‐related parameters (HBsAg, anti‐HBc, anti‐HBs, HBV‐DNA) liver‐related parameters (e.g., liver stiffness measurement (LSM), hepatic encephalopathy, oesophageal varices, ascites), laboratory parameters (e. g. MELD score, FIB‐4 score) as well as comorbidities. These variables were collected to adjust for sociodemographic and lifestyle‐related factors known to influence liver disease progression, as well as for HBV serological markers required to accurately characterize infection status and to isolate the effect of anti‐HBc positivity. Metabolic comorbidities were considered due to their independent and potentially synergistic contribution to liver disease progression, hepatocellular carcinoma development, and liver‐related mortality, and their potential to confound the association between anti‐HBc positivity and liver‐related outcomes. Age was stratified into < 50 years, 50–70 years, and > 70 years. This stratification was predefined in the DHC registry at the time of cohort establishment and has been consistently applied in previous publications using this registry. Alcohol consumption was also stratified according to the definitions of the DHC registry as no alcohol intake, moderate alcohol intake (≤ 40 g/day for men and ≤ 30 g/day for women), and high alcohol intake (> 40 g/day for men and > 30 g/day for women). These categories were therefore retained to ensure internal consistency and longitudinal comparability. The FIB‐4 index was categorized using the established cut‐off values of < 1.45 (low probability of advanced fibrosis), 1.45–3.25 (intermediate probability), and > 3.25 (high probability of advanced fibrosis). The diagnosis of cirrhosis was established on a composite score, based on the presence of cirrhosis‐related complications, such as ascites, or confirmed through histology, ultrasound, liver stiffness via FibroScan (> 12.5 kPa), the aspartate aminotransferase (AST)/platelet ratio index (APRI) (> 2.0), or the FIB‐4 index (> 3.25). Only patients with complete baseline (BL) data and follow‐up data at 12 or 24 weeks were included in the study. Statistical analyses included data from patients who initiated antiviral treatment between February 1, 2014, and July 14, 2018. Patients who had undergone organ transplantation, were HBsAg positive, or were coinfected with HIV were not included in the analysis. In addition, we performed a subanalysis restricted to patients with cirrhosis, defined according to the composite score (as described above), or advanced fibrosis (LSM > 10 kPa), including 2008 patients.

### 2.2 Statistical Analysis

The data cut‐off was on 14 July, 2023. Statistical analyses were performed using SPSS (IBM Corp. Released 2023. IBM SPSS Statistics for Windows, Version 26.0. Armonk, NY: IBM Corp.). Continuous variables were presented as median values (range: minimum to maximum). Categorical variables were described as percentages. To identify potential predictors of LRE and de novo HCC, univariate and multivariable logistic regression analyses were performed based on predefined hypotheses. We considered three different outcomes: (1) all LRE, including HCC, (2) all LRE excluding HCC, and (3) HCC alone. Only newly occurring events were considered LRE. LRE included a ≥ 3‐point increase in MELD score, new‐onset complications (oesophageal variceal bleeding, ascites, encephalopathy), liver transplantation, and death, either with or without de novo HCC, as well as isolated de novo HCC, diagnosed at follow‐up after 12/24 months or later post‐SVR. To assess the longitudinal incidence of these endpoints, Kaplan–Meier survival analyses with matched‐pair comparisons were conducted. Statistical significance was evaluated using the log‐rank test. A 95% confidence interval (CI) and a two‐sided *p*‐value < 0.05 were applied.

## 3 Results

### 3.1 Characteristics of Study Population

**FIGURE 1:**
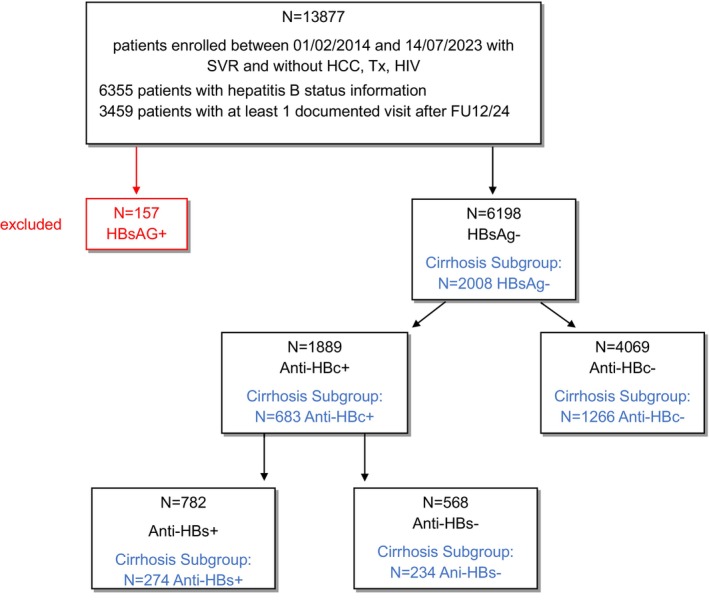
Patient flow chart. Cirrhosis subgroup consisting of patients with cirrhosis (definition is based on a composite score, incorporating the presence of cirrhosis‐related complications (e.g., ascites) or confirmation through histology, ultrasound, liver stiffness measurement (LSM) via FibroScan (> 12.5 kPa), the AST/platelet ratio index (APRI > 2.0), or the FIB‐4 index (> 3.25)) and patients with advanced fibrosis (LSM > 10 kPa). Anti‐HBc, Antibody to hepatitis B core antigen; FU, Follow‐up; HBsAg, Hepatitis B surface antigen; HCC, Hepatocellular carcinoma; HIV, Human immunodeficiency virus; SVR, Sustained virological response; Tx, Transplantation.

Only patients with sustained virologic response (SVR) were included in the present analysis. Individuals with a history of HCC, HIV infection, prior liver transplantation or HBsAg positivity were excluded. Between February 1, 2014, and July 14, 2023, a total of 6198 patients with available HBV serology were enrolled. Among these, 4069 patients (66%) were anti‐HBc negative, and 1889 (30%) were anti‐HBc positive, indicating resolved HBV infection. Of the anti‐HBc positive patients, 782 (41%) were anti‐HBs positive, and 568 (30%) were anti‐HBs negative. Overall, cirrhosis was present in 2008 patients. The patient flow chart is depicted in Figure 1.

The study cohort was predominantly male (*n* = 3773; 61%). Among anti‐HBc positive individuals, the proportion of male patients was 67% (*n* = 1260). Within the cirrhosis‐only subcohort, 1258 patients (63%) were male; among anti‐HBc positive patients with cirrhosis, 445 (65%) were male. The median age of the overall study population was 51 years (range, 18–88), whereas the median age in the cirrhosis subanalysis was 57 years (range, 20–88). The mean body mass index (BMI) was 26 kg/m^2^ in the overall cohort and 27 kg/m^2^ in the cirrhosis subcohort. In the overall cohort, alcohol consumption, irrespective of quantity, was reported by 813 participants (13%), while 1532 patients (25%) reported regular coffee intake. In the cirrhosis subanalysis, 290 patients (14%) reported regular alcohol consumption irrespective of quantity, and 380 (19%) reported regular coffee intake. Among patients with cirrhosis, 246 (12.3%) had oesophageal varices, 67 (3.3%) had ascites, and 37 (1.8%) had a history of hepatic encephalopathy at the time of SVR.

When comparing anti‐HBc positive and anti‐HBc negative patients in the overall cohort, significant differences were observed with respect to sex (*p* < 0.001), age (*p* < 0.001), LSM (*p* < 0.001), presence of cirrhosis (*p* < 0.001), FIB‐4 score (*p* = 0.002), and platelet count (*p* < 0.001). In the cirrhosis subgroup, there was only a significant difference regarding age (*p* = 0.002). Additional demographic and clinical characteristics of the cohort are summarized in Table 1.

**TABLE 1:** Baseline characteristics of study population for the analysis of clinical endpoints — demographics.

Parameter	*N* (%)						*p*‐value	
	Total		HBsAg−/Anti‐HBc‐		HBsAg−/Anti‐HBc+		(Anti‐HBc + vs. Anti‐HBc‐)	
Demographics	Overall cohort	Cirrhosis subgroup	Overall cohort	Cirrhosis Subgroup	Overall Cohort	Cirrhosis Subgroup	Overall cohort	Cirrhosis Subgroup
Total	6198	2008	4069	1266	1889	683		
Female Sex	2420 (39%)	750 (37%)	1710 (42%)	489 (39%)	629 (33%)	238 (35%)	0.000	0.105
Age								
< 50 years	2627 (42%)	506 (25%)	1764 (43%)	307 (24%)	738 (39%)		0.000	0.002
50–70 years	3091 (50%)	1229 (61%)	1963 (48%)	761 (60%)	1028 (54%)		0.000	0.002
> 70 years	480 (8%)	273 (14%)	342 (8%)	198 (16%)	123 (6.5%)		0.000	0.002
BMI (kg/m^2^) Mean ± SD	26 ± 4.9	27 ± 5	26 ± 5.0	27 ± 4.9	26 ± 4.9	27 ± 5.1		
Alcohol consumption (*n* = 6008)	813 (13%)	290 (14%)	535 (13%)	176 (14%)	253 (14%)	106 (16%)	0.375	0.209
Coffee consumption (*n* = 1891)	1532 (25%)	380 (19%)	1010 (25%)	255 (20%)	439 (23%)	110 (16%)	0.980	0.698
Liver‐related parameters								
LSM	*n* = 2871 (46%)	*n* = 1111 (55%)	*n* = 1923 (47%)	*n* = 702 (56%)	*n* = 870 (46%)	*n* = 383 (56%)		
Mean ± SD	11 ± 10.1	18.8 ± 12.5	10.3 ± 9.4	18 ± 11.8	12.5 ± 11.5	20.2 ± 13.5	0.000	0.012
LSM < 7 kPa	1222 (48%)	89 (9%)	871 (51%)	61 (10%)	319 (41%)	26 (8%)		
LSM 7–9.4 kPa	434 (17%)	65 (7%)	288 (17%)	47 (8%)	131 (17%)	16 (5%)		
LSM 9.5–12.4 kPa	282 (11%)	234 (23%)	176 (10%)	148 (24%)	97 (13%)	77 (22%)		
LSM ≥ 12.5	615 (24%)	615 (61%)	374 (22%)	374 (59%)	229 (30%)	229 (66%)		
Cirrhosis (according to composite score)	1874 (30%)	1874 (93%)	1184 (29%)	1184 (94%)	635 (34%)	635 (93%)	0.000	
Oesophageal varices	249 (4%)	246 (12%)	151 (4%)	149 (12%)	93 (5%)	92 (14%)	0.024	0.224
Ascites	67 (1%)	67 (3%)	39 (1%)	39 (3%)	27 (1%)	27 (4%)	0.109	0.292
Hepatic encephalopathy	37 (0.6%)	37 (2%)	20 (0.5%)	20 (2%)	16 (0.8%)	16 (2%)	0.106	0.220
MELD‐Score	*n* = 2099	*n* = 758						
Mean ± SD	7.4 ± 2.5	8.1 ± 2.6	7.4 ± 2.6	8.1 ± 2.7	7.4 ± 2.2	8.1 ± 2.4		
FIB‐4‐Score	*n* = 5802	*n* = 1892						
Mean ± SD	2.0 ± 2.2	3.6 ± 3.3	1.9 ± 2.2	3.5 ± 3.4	2.2 ± 2.2	3.7 ± 3.1	0.002	0.467
FIB‐4‐Score < 1.45	3055 (53%)	349 (18%)	2066 (55%)	231 (19%)	865 (48.%)	109 (17%)		
FIB‐4‐Score 1.45–3.25	1972 (34%)	768 (41%)	1232 (33%)	473 (40%)	656 (37%)	270 (42%)		
FIB‐4‐Score ≥ 3.25	775 (13%)	775 (41%)	488 (13%)	488 (41%)	266 (15%)	266 (41%)		
Laboratory parameters								
Aspartate aminotransferase (U/l)	*n* = 5839	*n* = 1899						
Mean ± SD	41 ± 38	55 ± 54	40 ± 35	54 ± 49	42 ± 42	56 ± 61	0.072	0.355
Alanine aminotransferase (U/l)	*n* = 6123	*n* = 1986						
Mean ± SD	44 ± 55	53 ± 66	44 ± 54	51 ± 62	44 ± 55	54 ± 71	0.565	0.485
Gamma glutamyltransferase (U/l)	*n* = 6104	*n* = 1983						
Mean ± SD	58 ± 99	93 ± 147	56 ± 96	91 ± 147	62 ± 105	95 ± 148	0.024	0.582
Akaline phosphatase (U/l)	*n* = 5286	*n* = 1756						
Mean ± SD	81 ± 33	95 ± 42	80 ± 33	95 ± 42	82 ± 32	96 ± 40		
Bilirubin (mg/dl)	*n* = 5929	*n* = 1944						
Mean ± SD	0.6 ± 0.4	0.8 ± 0.5	0.6 ± 0.4	0.8 ± 0.5	0.6 ± 0.4	0.8 ± 0.6		
Albumin (g/l)	*n* = 3593	*n* = 1287						
Mean ± SD	42 ± 6	41 ± 7	42 ± 7	41 ± 8	42 ± 6	41 ± 6		
Platelets (thousand/μl)	*n* = 6100	*n* = 1984						
Mean ± SD	209 ± 74	157 ± 71	211 ± 74	159 ± 74	203 ± 74	154 ± 67	0.000	0.166
International normalized ratio	*n* = 2164	*n* = 772						
Mean ± SD	1.1 ± 0.3	1.1 ± 0.3	1.1 ± 0.3	1.1 ± 0.3	1.1 ± 0.2	1.1 ± 0.2		
HbA1c (%)	*n* = 1253	*n* = 443						
Mean ± SD	5.6 ± 0.9	5.7 ± 1.1	5.7 ± 0.9	5.7 ± 1.0	5.6 ± 0.9	5.7 ± 1.2		
Comorbidites								
Tumour disease*	305 (5%)	165 (8%)	193 (5%)	105 (8%)	104 (6%)	55 (8%)	0.315	0.803
Dyslipidaemia	135 (2%)	48 (2%)	86 (2%)	29 (2%)	47 (3%)	19 (3%)	0.396	0.541
Metabolic syndrome	54 (0.9%)	22 (1%)	37 (0.9%)	15 (1%)	16 (0.8%)	6 (0.9%)	0.883	0.649
Diabetes mellitus	576 (9%)	333 (17%)	379 (9%)	213 (17%)	178 (9%)	111 (16%)	0.886	0.799
Type I	51 (9%)	27 (8%)	33 (9%)	16 (8%)	16 (9.0%)	10 (9%)		
Type II	525 (91%)	306 (92%)	346 (91%)	197 (93%)	162 (91%)	101 (91%)		

*Note:* Cirrhosis definition is based on a composite score, incorporating the presence of cirrhosis‐related complications (e.g., ascites) or confirmation through histology, ultrasound, liver stiffness measurement (LSM) via FibroScan (> 12.5 kPa), the AST/platelet ratio index (APRI > 2.0), or the FIB‐4 index (> 3.25). * Malignancies (including solid tumors, malignant lymphomas (Hodgkin and Non‐Hodgkin), all forms of leukemia and organ‐based tumors). Abbreviations: Anti‐HBc, antibody to hepatitis B core antigen; BMI, body mass index; FIB‐4‐Score, Fibrosis‐4 score; HbA1c, Glycated Haemoglobin (Haemoglobin A1c); HBsAg, hepatitis B surface antigen; M, mean; SVR, sustained virological response; SD, standard deviation; LSM, liver stiffness measurement; MELD‐Score, Model for End‐Stage Liver Disease score.

**TABLE 2a:** Regression analysis – overall cohort.

Parameter	LRE including HCC (*n* = 177)		LRE exluding HCC (*n* = 143)		HCC (*n* = 64)	
	Univariate	Multivariate	Univariate	Mutivariate	Univariate	Multivariate
Male Sex	1.33 (0.97–1.82)	**1.52 (1.08–2.14)**	1.41 (0.99–2.00)	**1.66 (1.13–2.43)**	0.91 (0.53–1.58)	0.93 (0.52–1.67)
Age 50–70 vs. < 50	**2.02 (1.38–2.95)**	1.41 (0.95–2.10)	**1.72 (1.14–2.58)**	1.25 (0.81–1.92)	**5.54 (2.17–14.11)**	**3.25 (1.25–8.44)**
Age > 70 vs. < 50	**4.10 (2.54–6.761)**	**2.53 (1.50–4.30)**	**3.73 (2.23–6.22)**	**2.64 (1.50–4.65)**	**7.52 (2.50–22.60)**	2.76 (0.85–8.98)
Liver Stiffness	**1.05 (1.03–1.06)**	—	**1.05 (1.03–1.06)**	—	**1.05 (1.02–1.08)**	—
Cirrhosis	**4.92 (3.54–6.83)**	**4.00 (2.82–5.66)**	**4.04 (2.83–5.74)**	**3.34 (2.29–4.86)**	**13.16 (5.92–29.28)**	**10.14 (4.48–22.93)**
Esophageal varices	**9.41 (6.39–13.85)**	—	**9.64 (6.37–14.59)**	—	**8.68 (4.63–16.29)**	—
Ascites	**11.15 (6.14–20.25)**	—	**11.86 (6.39–22.01)**	—	**7.59 (2.89–19.98)**	—
Hepatic encephalopathy	3.12 (0.91–10.70)	—	**3.97 (1.16–13.64)**	—	3.16 (0.42–24.00)	—
Anti‐HBc positivity	**1.46 (1.07–2.00)**	1.38 (1.00–1.90)	**1.47 (1.04–2.08)**	1.40 (0.98–1.99)	1.37 (0.77–2.41)	1.21 (0.68–2.16)
alcohol ≤ 40 g/day in men or ≤ 30 g/day in women vs. no alcohol consumption	**0.49 (0.25–0.98)**	—	0.56 (0.27–1.16)	—	0.36 (0.09–1.50)	—
Tumor^a^	**11.17 (7.88–15.83)**	—	**4.19 (2.72–6.47)**	—	521768555.49	—
Diabetes^b^	**2.72 (1.90–3.90)**	**1.57 (1.06–2.33)**	**2.35 (1.55–3.55)**	1.49 (0.96–2.30)	**2.47 (1.28–4.75)**	1.22 (0.61–2.44)
Metabolic syndrome	2.51 (0.87–7.23)	—	**3.16 (1.10–9.12)**	—	0.00 (0.00–0.00)	—

*Note:* Statistically significant results highlighted in bold; Odds ratio is given, with 95% confidence interval in parenthesis. Cirrhosis definition is based on a composite score, incorporating the presence of cirrhosis‐related complications (e.g., ascites) or confirmation through histology, ultrasound, liver stiffness measurement (LSM) via FibroScan (> 12.5 kPa), the AST/platelet ratio index (APRI > 2.0), or the FIB‐4 index (> 3.25).

Abbreviations: Anti‐HBc, antibody to hepatitis B core antigen; HCC, hepatocellular carcinoma; LRE, liver‐related events.

^a^ Malignancies (including solid tumors, malignant lymphomas (Hodgkin and Non‐Hodgkin), all forms of leukemia and organ‐based tumors).

^b^ Diabetes Type I and II.

### 3.2 Outcomes

To investigate the effect of a past HBV infection on LRE (defined as an increase in MELD score by ≥ 3 points, new‐onset events such as oesophageal variceal bleeding, ascites, encephalopathy, liver transplantation, and/or death, including de novo HCC, defined as diagnosis at FU12/24 or later after SVR, or excluding de novo HCC) and the development of HCC alone, we performed univariate and multivariate analyses and constructed Kaplan–Meier curves. The median time of follow‐up was 2.5 years (range 0.04–8.01); additionally, the mean follow‐up duration was 3.0 ± 1.9 years.

During follow‐up, a MELD score increase of ≥ 3 points occurred in 49 patients in the overall cohort, including 16 anti‐HBc positive individuals. In the cirrhosis subgroup, 30 patients showed a MELD increase of ≥ 3 points, of whom 11 were anti‐HBc positive. In the overall cohort, oesophageal variceal bleeding occurred in 18 patients (7 anti‐HBc positive), new‐onset ascites in 21 patients (7 anti‐HBc positive), and hepatic encephalopathy in 17 patients (9 anti‐HBc positive). Four patients underwent liver transplantation (one anti‐HBc positive), and 92 patients died during follow‐up (42 anti‐HBc positive), with 28 deaths being liver‐related. In the cirrhosis subgroup, variceal bleeding occurred in 17 patients (7 anti‐HBc positive), ascites in 18 (6 anti‐HBc positive), and hepatic encephalopathy in 16 (9 anti‐HBc positive). Liver transplantation was performed in four patients (one anti‐HBc positive), and 61 patients died (30 anti‐HBc positive).

Overall, 177 patients in the overall cohort and 125 patients in the cirrhosis subgroup experienced at least one LRE during follow‐up, including de novo HCC; among these, 70 anti‐HBc positive patients were identified in the overall cohort and 54 in the cirrhosis subgroup. After exclusion of de novo HCC, 143 patients in the overall cohort and 96 in the cirrhosis subgroup experienced an LRE, including 57 and 42 anti‐HBc positive patients. De novo HCC developed in 64 patients in the overall cohort and in 57 patients in the cirrhosis subgroup, including 20 and 18 anti‐HBc–positive individuals.

### 3.3 Factors Associated With Liver‐Related Outcomes

To identify predictors of LRE, we performed univariate and multivariate logistic regression analyses for the overall cohort and the cirrhosis subgroup. The univariate analysis included a comprehensive set of potential risk factors selected a priori based on their established or plausible association with liver disease progression, portal hypertension, HCC, and liver‐related mortality, as well as their potential to confound the association between anti‐HBc positivity and LRE. These variables comprised sex, age, BMI, alcohol and coffee consumption, HBV serological markers (HBsAg, anti‐HBc, anti‐HBs), markers of liver disease severity (LSM, cirrhosis, hepatic encephalopathy, oesophageal varices, ascites), malignancies (including solid tumours, malignant lymphomas such as Hodgkin and Non‐Hodgkin lymphoma, and all forms of leukaemia, including lymphatic and myeloid leukaemias, as well as organ‐based tumours classified as lymphomas), and other comorbidities (diabetes, dyslipidaemia, metabolic syndrome, inflammatory joint diseases). When we refer to “diabetes” in this text, we include all patients with any form of diabetes, including both type 1 and type 2 diabetes. Variables with predefined clinical relevance for this research or statistical significance in the univariate analysis were then considered in the multivariate model. The final multivariate logistic regression included the following covariates: sex, age, anti‐HBc, cirrhosis, and diabetes mellitus. A detailed overview of all variables included in the regression analysis, along with the corresponding results, is provided in Tables S1–S6.

**TABLE 2b:** Regression analysis – cirrhosis subgroup.

Parameter	LRE including HCC (*n* = 125)		LRE exluding HCC (*n* = 96)		HCC (*n* = 57)	
	Univariate	Multivariate	Univariate	Mutivariate	Univariate	Multivariate
Male Sex	1.11 (0.76–1.62)	1.19 (0.79–1.78)	1.21 (0.78–1.86)	1.33 (0.84–2.11)	0.85 (0.47–1.55)	0.89 (0.48–1.67)
Age 50–70 vs. < 50	1.22 (0.75–1.97)	1.12 (0.69–1.84)	1.08 (0.64–1.84)	1.02 (0.59–1.75)	2.40 (0.93–6.24)	2.19 (0.83–5.76)
Age > 70 vs. < 50	1.64 (0.91–2.97)	1.56 (0.83–2.96)	1.47 (0.76–2.83)	1.56 (0.77–3.15)	2.78 (0.92–8.42)	2.14 (0.66–6.98)
Liver Stiffness	**1.04 (1.02–1.06)**	—	**1.01 (1.02–1.06)**	—	1.02 (0.99–1.06)	—
Cirrhosis	**4.89 (1.19–20.12)**	**4.63 (1.12–19.14)**	3.63 (0.89–15.00)	3.48 (0.84–14.46)	67001294.47	62700054.47
Esophageal varices	**4.88 (3.19–7.45)**	—	**5.63 (3.54–8.97)**	—	**3.41 (1.77–6.56)**	—
Ascites	**5.66 (3.08–10.42)**	—	**6.57 (3.48–12.42)**	—	**3.06 (1.15–8.13)**	—
Hepatic encephalopathy	1.52 (0.44–5.23)	—	2.09 (0.60–7.23)	—	3.16 (0.42–24.00)	—
Anti‐HBc positivity	**1.48 (1.01–2.15)**	**1.53 (1.04–2.25)**	1.48 (0.97–2.26)	**1.54 (1.00–2.37)**	1.26 (0.68–2.33)	1.29 (0.69–2.40)
alcohol ≤ 40 g/day in men or ≤ 30 g/day in women vs. no alcohol consumption	0.50 (0.21–1.15)	—	0.56 (0.22–1.41)	—	0.46 (0.11–1.92)	—
Tumor^a^	**10.34 (6.77–15.92)**	—	**3.35 (2.02–5.55)**	—	908704901.41	—
Diabetes^b^	**3.23 (1.01–10–31)**	1.35 (0.86–2.12)	1.47 (0.90–2.41)	1.30 (0.78–2.17)	1.30 (0.64–2.67)	1.04 (0.49–2.22)
Metabolic syndrome	3.23 (1.01–10.31)	—	**4.37 (1.36–13.99)**	—	0.00 (0.00–0.00)	—

*Note:* Statistically significant results highlighted in bold; Odds ratio is given, with 95% confidence interval in parenthesis. Cirrhosis definition is based on a composite score, incorporating the presence of cirrhosis‐related complications (e.g., ascites) or confirmation through histology, ultrasound, liver stiffness measurement (LSM) via FibroScan (> 12.5 kPa), the AST/platelet ratio index (APRI > 2.0), or the FIB‐4 index (> 3.25).

Abbreviations: Anti‐HBc, antibody to hepatitis B core antigen; HCC, hepatocellular carcinoma; LRE, liver‐related events.

^a^ Malignancies (including solid tumors, malignant lymphomas (Hodgkin and Non‐Hodgkin), all forms of leukemia and organ‐based tumors).

^b^ Diabetes Type I and II.

#### 3.3.1 Liver‐Related Events Including de Novo HCC

In the overall cohort, univariate analysis showed that anti‐HBc positivity was significantly associated with an increased risk of LRE, including HCC (OR 1.46, 95% CI 1.07–2.00, *p* = 0.017). Additional risk factors included age (*p* < 0.001; OR 2.02, 95% CI 1.38–2.95 for 50–70 years, OR 4.10, 95% CI 2.54–6.61 for > 70 years), elevated LSM (OR 1.05, 95% CI 1.01–1.06, *p* < 0.001), cirrhosis (OR 4.92, 95% CI 3.54–6.83, *p* < 0.001) and its complications, including oesophageal varices (OR 9.40, 95% CI 6.39–13.85, *p* < 0.001) and ascites (OR 11.15, 95% CI 6.14–20.25, *p* < 0.001), as well as malignancies (OR 11.17, 95% CI 7.88–15.83, *p* < 0.001) and diabetes (OR 2.61, 95% CI 1.81–3.77, *p* < 0.001). Interestingly, moderate alcohol consumption (< 40 g/day in men and < 30 g/day in women) appeared to have a protective effect (OR 0.50, 95% CI 0.25–0.98, *p* = 0.044).

In multivariate analysis of the overall cohort, male sex (OR 1.52, 95% CI 1.08–2.14, *p* = 0.017), age > 70 years (OR 2.53, 95% CI 1.50–4.30, *p* = 0.001), cirrhosis (OR 4.00, 95% CI 2.82–5.66, *p* < 0.001), and diabetes (OR 1.57, 95% CI 1.06–2.33, *p* = 0.024) were confirmed as independent risk factors for LRE, including de novo HCC.

In the subgroup analysis restricted to patients with cirrhosis, anti‐HBc positivity remained a significant predictor of LRE including de novo HCC in both univariate (OR 1.48, 95% CI 1.01–2.15, *p* = 0.044) and multivariate analyses (OR 1.53, 95% CI 1.04–2.26, *p* = 0.030) (Table 2). Within this cirrhosis cohort, elevated LSM (*p* < 0.001) and cirrhosis‐related complications—including oesophageal varices (OR 9.40, 95% CI 6.39–13.85, *p* < 0.001) and ascites (OR 11.15, 95% CI 6.14–20.25, *p* < 0.001)—were associated with increased risk in univariate models. Malignancies (OR 11.17, 95% CI 7.88–15.83, *p* < 0.001), diabetes (OR 2.61, 95% CI 1.81–3.77, *p* < 0.001), and metabolic syndrome (OR 1.43, 95% CI 1.01–2.03, *p* = 0.047) also showed associations in univariate analysis. In multivariate analysis, however, cirrhosis‐related features and LSM remained the dominant independent predictors of LRE (cirrhosis *p* = 0.035, LSM *p* < 0.001) (Table 2).

#### 3.3.2 Liver‐Related Events Excluding de Novo HCC

When HCC was excluded, anti‐HBc positivity was still significantly associated with the occurrence of LRE in the overall cohort (OR 1.47, 95% CI 1.94–2.08, *p* = 0.028). In addition, older age (50–70 years: OR 1.72, 95% CI 1.14–2.58, *p* < 0.001; > 70 years: OR 3.73, 95% CI 2.23–6.22, *p* < 0.001), increased liver stiffness measurement (OR 1.047, 95% CI 1.031–1.063, *p* < 0.001) and cirrhosis (OR 2.04, 95% CI 2.83–5.74, *p* < 0.001), including its complications such as hepatic encephalopathy (*p* = 0.029), oesophageal varices (*p* < 0.001) and ascites (*p* < 0.001), were also significantly associated with this outcome. Furthermore, malignancies (*p* < 0.001), diabetes (*p* < 0.001), and metabolic syndrome (*p* = 0.033) showed to be risk factors for LRE excluding HCC.

In multivariable analysis, male sex was an independent risk factor (OR 1.66, 95% CI 1.13–2.43, *p* = 0.009) in the overall cohort. Advanced age (> 70 years; OR 2.64, 95% CI 1.50–4.65, *p* = 0.001) and cirrhosis (OR 3.34, 95% CI 2.29–4.86, *p* < 0.001) were also confirmed as independent predictors of LRE excluding HCC (Table 2).

In the cirrhosis subgroup analysis, anti‐HBc positivity was not significantly associated with an increased risk of LRE in univariate analysis (*p* = 0.07; Table 2). In contrast, higher LSM (*p* < 0.001), signs of portal hypertension (oesophageal varices (*p* < 0.001), ascites (*p* < 0.001)), malignancies (*p* < 0.001), and metabolic syndrome (*p* = 0.013) were significantly associated with LRE in univariate analysis.

In multivariable analysis of the subgroup, anti‐HBc positivity was the only factor associated with an increased risk of LRE excluding HCC (Table 2).

#### 3.3.3 De Novo HCC

When analysing the occurrence of HCC as a single endpoint in the overall cohort, anti‐HBc positivity was no significant risk factor in the univariate analysis (*p* = 0.282). In contrast, increasing age was strongly associated with HCC, both in patients aged 50–70 years (OR 5.38, 95% CI 2.17–14.11, *p* = 0.011) and in those older than 70 years (OR 7.52, 95% CI 2.50–22.60, *p* < 0.001). Higher LSM (OR 1.05, 95% CI 1.02–1.08, *p* < 0.001), cirrhosis (OR 13.16, 95% CI 5.92–29.28, *p* < 0.001), and cirrhosis‐related complications (oesophageal varices (*p* < 0.001), ascites (*p* < 0.001)) were also significantly associated with HCC occurrence. In addition, diabetes mellitus was significantly associated with HCC development (*p* = 0.007) (Table 2).

In multivariable analysis, age 50–70 years (*p* = 0.016) and cirrhosis (*p* < 0.001) remained independently associated with HCC.

Regarding the development of de novo HCC in the cirrhosis subgroup, anti‐HBc positivity was also not significantly associated in univariate analysis (*p* = 0.46) (Table 2). The only significant risk factors were signs of portal hypertension such as oesophageal varices (OR 3.41, 95% CI 1.77–6.56, *p* < 0.001) and ascites (OR 3.06, 95% CI 1.77–6.56, *p* = 0.25).

In multivariate analysis of the cirrhosis group, no factor was significantly associated with HCC (Table 2).

Taken together, anti‐HBc positivity was associated with LRE when composite outcomes were considered, whereas no association was observed with the isolated development of de novo HCC. The prognostic relevance of anti‐HBc positivity was more evident in patients with cirrhosis. In contrast, established markers of disease severity, including cirrhosis, LSM, and features of portal hypertension, remained the predominant determinants of HCC risk.

### 3.4 Kaplan–Meier Analysis

To further explore the temporal dimension of LRE and complement the findings from the logistic regression models, Kaplan–Meier survival analyses were performed for the overall cohort and the cirrhosis subgroup focusing on the influence of HBV serological marker, particularly anti‐HBc status, on event‐free survival.

**FIGURE 2:**
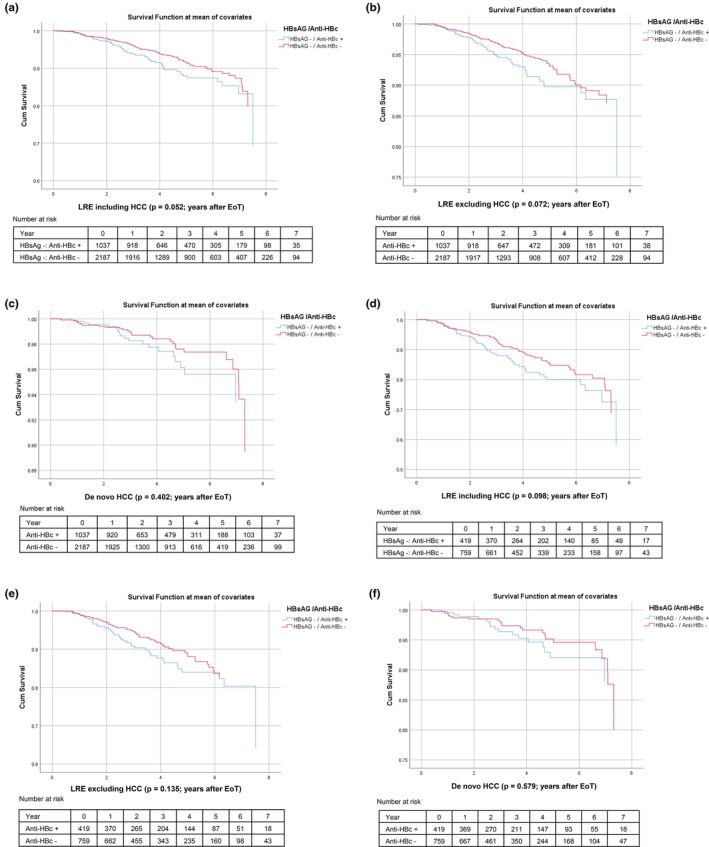
(a) KPK liver‐related events including de novo‐HCC—overall cohort. Clinical endpoints in the German Hepatitis C Registry. HBV‐serological markers at baseline were tested for their predictive value regarding clinical endpoints. Kaplan–Meier curves demonstrating the rate of liver‐related events including de novo hepatocellular carcinoma in HBsAG negative patients with SVR after HCV‐infection, who are anti‐HBc positive versus negative. (b) KPK liver‐related events excluding de novo‐HCC—overall cohort. (c) KPK HCC—overall cohort. (d) KPK liver‐related events including de novo‐HCC–cirrhosis subgroup. (e) KPK liver‐related events excluding de novo‐HCC—cirrhosis subgroup. (f) KPK HCC–cirrhosis subgroup. Anti‐HBc, Antibody to hepatitis B core antigen; EoT, End of treatment; HBsAG, Hepatitis B surface antigen; HCC, Hepatocellular carcinoma; LRE, Liver‐related events.

In Kaplan–Meier analyses of the overall cohort, no significant differences in event‐free survival were observed between anti‐HBc positive and anti‐HBc negative patients, either for LRE including de novo HCC (*p* = 0.052), LRE excluding HCC (*p* = 0.072), or de novo HCC as a single endpoint (*p* = 0.402) (Figure 2a–c).

Similarly, in Kaplan–Meier analyses restricted to the cirrhosis subgroup, anti‐HBc positivity was not significantly associated with event‐free survival, irrespective of whether LRE included HCC (*p* = 0.098), excluded HCC (*p* = 0.135), or were limited to de novo HCC (*p* = 0.579) (Figure 2a–f). Additional subanalyses focusing on anti‐HBc positive patients are provided in the Supporting Information (Figure S1–S6).

Taken together, Kaplan–Meier analyses did not demonstrate a significant impact of anti‐HBc positivity on time‐to‐event outcomes, suggesting that it has limited prognostic relevance.

## 4 Discussion

The extent to which prior exposure to HBV contributes to liver‐related complications in patients cured of HCV infection remains a subject of ongoing debate. To address this question, we analysed a large cohort of 6198 patients enrolled in the German Hepatitis C Registry—a prospective, multicentre registry that includes both academic referral centres and real‐world clinical settings, such as office‐based physicians managing HCV‐infected individuals [11]. The registry provides a high‐quality dataset, supported by rigorous central data validation and both on‐site and remote monitoring procedures. This analysis specifically aimed to investigate the potential impact of resolved HBV infection, as indicated by anti‐HBc positivity, on the progression of liver disease and the risk of HCC.

For this mainly Caucasian cohort, our analyses showed that in patients with cirrhosis, anti‐HBc positivity was linked to a more than 1.5‐fold higher likelihood of LRE including and excluding HCC after a median follow‐up time of 2.5 years (range 0.04–8.01) after SVR. When considering de novo HCC alone, anti‐HBc positivity was not identified as a risk factor. Instead, markers of advanced liver disease, such as oesophageal varices and ascites, were more strongly associated with HCC risk in univariate analysis. Overall, the relevance of anti‐HBc positivity appears inconsistent, as Kaplan–Meier survival analyses showed no significant association with anti‐HBc status. Visual inspection of the Kaplan–Meier curves suggests that anti‐HBc positive patients, both in the overall cohort and in the cirrhosis subgroup, consistently showed slightly lower survival probabilities compared with anti‐HBc negative patients, regardless of the endpoint considered. Nonetheless, in our large cohort with a median follow‐up of 2.5 years, no statistically significant associations were observed (Figure 2a–f).

These findings reflect the heterogeneity also reported in previous studies, such as the 2016 meta‐analysis that demonstrated variable HCC risks depending on anti‐HBc status [12].

The role of previous exposure to HBV for the risk to develop clinical complications of liver disease has not been studied in detail in patients who have been cured from chronic hepatitis C. In a meta‐analysis, resolved HBV infection was significantly associated with chronic liver disease [13] but this study did not include HCV patients after DAA therapy. This highlights the relevance of our research question, while also reflecting the heterogeneity of available data and the complexity of the issue. The recently published EASL recommendations for the follow‐up after HCV cure do not consider the anti‐HBc status [14]. This is largely consistent with our findings, although the association between anti‐HBc positivity and composite liver‐related endpoints in regression analysis observed in our study should not be disregarded. The precise clinical relevance of anti‐HBc positivity should therefore be further evaluated in larger cohorts with longer follow‐up periods. With regard to overall survival, however, we did not observe an association with previous HBV infection. Nevertheless, clinicians should be aware that HBV reactivation, although rare in immunocompetent individuals, can still occur under immunosuppressive therapies such as chemotherapy or biologics, particularly in anti‐HBc positive patients without anti‐HBs, as reported in the setting of rituximab‐containing lymphoma treatments [15].

Beyond anti‐HBc status, metabolic comorbidities such as diabetes and metabolic syndrome were partially associated with LRE in our overall cohort. Within the German Hepatitis C‐Registry, we have previously shown that elevated liver enzymes after HCV cure were associated with metabolic features [16]. Age, male sex, and cirrhosis have been identified as well‐established risk factors for liver disease progression in cohorts from North America [17], Asia [18], Europe [19, 20] as well as in specific populations such as HIV positive individuals [21], and were likewise significant risk factors in our overall cohort. However, in our cirrhosis subgroup analysis, these factors were no longer significantly associated with outcomes; instead, markers of advanced liver disease, reflecting portal hypertension, such as ascites and oesophageal varices, emerged as the leading risk factors.

HCC remains one of the most serious complications in patients with advanced liver disease. It is well established that active HBV infection is strongly associated with the development of HCC [22, 23, 24]. In contrast, and somewhat surprisingly, anti‐HBc positivity was not identified as a risk factor for HCC in our analyses. This finding is partially in contrast with the results of a 2016 meta‐analysis, which demonstrated that the risk of HCC was significantly higher in patients with chronic liver disease who were anti‐HBc positive [12]. A systematic review from 2014 revealed a notably higher prevalence of occult hepatitis B in patients with HCC, regardless of the presence of HCV infection, compared to control populations without HCC [25] and a meta‐analysis conducted in 2012 indicated that occult HBV infection may act as a cofactor in the development of HCV‐related HCC [26]. Mechanistically, data suggest that oncogenic properties of HBV may persist even in the setting of occult infection, including low‐level viral replication and chronic liver inflammation [10]. A study in patients with cryptogenic HCC found occult hepatitis B in up to 73% of cases [27], underscoring the potential clinical relevance of resolved infection despite HBsAg negativity. In experimental models, such as in woodchucks chronically infected with WHV, a virus closely related to HBV, occult infection was also associated with HCC development in up to 20% of animals within five years [28]. These findings suggest a potential biological plausibility that should not be underestimated. At this point, it should be emphasized that our analysis does not allow conclusions regarding occult hepatitis B, as comprehensive HBV DNA data were not available. The status of resolved HBV infection was therefore determined based on anti‐HBc positivity. Still, long‐term follow‐up after HCV cure with DAAs did not consider anti‐HBc in many studies as it was not associated with an increased risk of HCC after HCV eradication [29].

One of the strengths of our study is the large cohort size, which was prospectively followed in a multicentre design. The inclusion of both physicians from university hospitals and private practices provides a broad and representative sample, enhancing the generalizability of our findings. Additionally, central monitoring was implemented to ensure consistency in data collection and protocol adherence across sites. When it comes to limitations, missing values were present in some key variables, and the absence of a centralized laboratory introduces variability in diagnostic testing and results. Moreover, despite the large cohort size, the follow‐up period was relatively short. Among anti‐HBc positive patients, HBV DNA was investigated in only a small subset, which limited the utility of differentiating between HBV DNA positive and HBV DNA negative individuals in this group. Consequently, we refrained from classifying patients as having occult HBV infection. Instead, all anti‐HBc positive patients were classified as having resolved HBV infection, in accordance with established serological definitions. Future studies should incorporate systematic HBV DNA testing in anti‐HBc positive individuals to enable a more accurate virological classification. In addition, longitudinal assessments of HBV DNA would be essential to better capture viral dynamics over time, including low‐level persistence and potential reactivation as well as long‐term risks associated with past HBV infection, particularly with regard to HCC and other LRE in patients with cured HCV infection.

## 5 Conclusion

Our study indicates that resolved HBV infection, as reflected by anti‐HBc positivity, is not consistently associated with LRE or overall survival in this predominantly Caucasian cohort after HCV cure. While anti‐HBc positivity showed associations with composite liver‐related outcomes in patients with cirrhosis, it did not emerge as a significant predictor of de novo HCC or event‐free survival in Kaplan–Meier analyses, irrespective of the endpoint considered. In contrast, established markers of advanced liver disease, such as features of portal hypertension, remained the primary determinants of adverse liver outcomes. These findings suggest that anti‐HBc status alone does not warrant modification of post‐SVR surveillance strategies. Future studies with longer follow‐up may help to further clarify the clinical significance of resolved HBV infection in this context.

## Funding

This work was supported by the German Center for Infection Research (DZIF). AbbVie Deutschland GmbH & Co. KG. Bristol‐Myers Squibb GmbH & Co. KGaA. Gilead Sciences GmbH. Janssen‐Cilag GmbH. MSD Sharp & Dohme GmbH. F. Hoffmann‐La Roche AG. Financial support for the German Hepatitis C‐Registry was obtained from the German Center for Infection Research (DZIF) and the companies AbbVie Deutschland GmbH & Co. KG (until 2024‐07‐14), Bristol‐Myers Squibb GmbH & Co. KGaA (until 2020‐07‐14), Gilead Sciences GmbH (until 2022‐07‐14), Janssen‐Cilag GmbH (until 2020‐07‐14), MSD Sharp & Dohme GmbH (until 2024‐07‐14), as well as Roche Pharma AG (until 2017‐07‐14). The maintenance of the database was supported by Gilead Sciences GmbH from (2022‐07‐15–2023‐07‐14).

## Ethics Statement

The study protocol was conducted in accordance with the Declaration of Helsinki and Good Clinical Practice guidelines approved by the institutional review board (Ethics Committee of Ärztekammer Westfalen‐Lippe; reference number 2014–395‐f‐S).

## Consent

Patients were required to provide written informed consent to be enrolled in the registry.

## Conflicts of Interest

Albrecht Stoehr has nothing to disclose. Peter Buggisch: served as speaker and/or consultant for AbbVie, Gilead, and MSD. Christine John has nothing to disclose. Ralph Link has nothing to disclose. Hartwig Klinker has nothing to disclose. Uta Merle: served as a speaker, consultant and/or advisory board member for Boehringer, CSL Behring, CytoSorbents Europe GmbH, Gilead, GSK, Ipsen, Falk Foundation, Univar, Microbiotica. Markus Cornberg: served as consultant for Abbvie, AiCuris, AstraZenenca, Gilead, GSK, MSD Sharp & Dohme, Roche and speaker for: Abbvie, Falk, Gilead. Christoph Sarrazin: served as a speaker, consultant, and/or advisory board member for AbbVie and Gilead. Thomas Berg: served as consultant and/or advisory board member for AbbVie, Alexion, Albireo, Bayer, Gilead, GSK, Eisai, Intercept, Ipsen, Madrigal, Mirum, Orphalan, Sequana Medical; speaker for AbbVie, Advance Pharma, Alexion, Bayer, Gilead, Eisai, Falk Foundation, Ipsen, MedUpdate GmbH, Orphalan; received research grants from AbbVie, Advanz, Gilead, Norgine, Orphalan, Sequana Medical. Heiner Wedemeyer: served as speaker for Gilead Sciences GmbH & Gilead Sciences Ltd. and Biotest AG; received grants from Biotest AG and Abbott Laboratories & Abbott Molecular Inc; served as consultant/advisory board for Gilead Sciences GmbH & Gilead Sciences Ltd., Abbott Laboratories & Abbott Molecular Inc., Bristol‐Myers‐Squibb, F. Hoffmann‐La Roche Ltd., GlaxoSmithKline Services Unlimited, Janssen, Roche Diagnostics International Ltd., and Vir Biotechnology Inc.

## Collaborators

Renate Heyne, Stefan Mauss, Hjordis Möller, Rainer Ullrich, Gerlinde Teuber, Stefan Christensen, Rainer Günther, Uwe Naumann, Jörg Petersen, Willibold Schiffelholz, Thomas Lutz, Karl‐Georg Simon, Heribert Knechten, Klaus H.W. Boeker, Ansgar Rieke, Jörn M. Schattenberg, Stefan Zeuzem, Nazifa Qurishi, Nikolaus Kordecki, Peter Geyer, Michael Priller, Johannes Roth, Martin Rössle, Holger Hinrichsen, Carsten Zamani, Norbert E. Lyonn, Andreas Weber, Pavel Khaykin, Heinz Hartmann, Dietrich Hüppe, Michael P. Manns, Ulrike Protzer, Peter Schirmacher.

## Supporting information


**Figure S1:** KPK liver‐related events including de novo‐HCC Overall Cohort.
**Figure S2:** KPK liver‐related events excluding de novo‐HCC Overall Cohort.
**Figure S3:** KPK de novo‐HCC Overall Cohort.
**Figure S4:** KPK liver‐related events including de novo‐HCC Cirrhosis Subgroup.
**Figure S5:** KPK liver‐related events excluding de novo‐HCC Cirrhosis Subgroup.
**Figure S6:** KPK de novo‐HCC Cirrhosis Subgroup.

**Table S1:** Liver‐related events including de novo HCC– Univariate Analysis Overall Cohort.
**Table S2:** Liver‐related events excluding de novo HCC—Univariate Analysis Overall Cohort.
**Table S3:** De novo HCC—Univariate Analysis Overall Cohort.
**Table S4:** Liver‐related events including de novo HCC—Univariate Analysis Cirrhosis Subgroup.
**Table S5:** Liver‐related events excluding de novo HCC—Univariate Analysis Cirrhosis Subgroup.
**Table S6:** De novo HCC—Univariate Analysis Cirrhosis Subgroup.

## Data Availability

The data that support the findings of this study are available on request from the corresponding author. The data are not publicly available due to privacy or ethical restrictions.
