# Supplementary material for: Resolved HBV Infection Is Not Associated With Liver‐Related Outcomes in Survival Analysis of Caucasians After HCV Cure
Source: Liver Int. 2026 Apr 30;46:e70620. doi: 10.1111/liv.70620 (PMC13130161; doi:10.1111/liv.70620)
Supplement: Supplementary file 1 — Figure S1: KPK liver‐related events including de novo‐HCC Overall Cohort. Figure S2: KPK liver‐related events excluding de novo‐HCC Overall Cohort. Figure S3: KPK de novo‐HCC Overall Cohort. Figure S4: KPK liver‐related events including de novo‐HCC Cirrhosis Subgroup. Figure S5: KPK liver‐related events excluding de novo‐HCC Cirrhosis Subgroup. Figure S6: KPK de novo‐HCC Cirrhosis Subgroup. [file LIV-46-0-s002.pptx]

## Slide 1
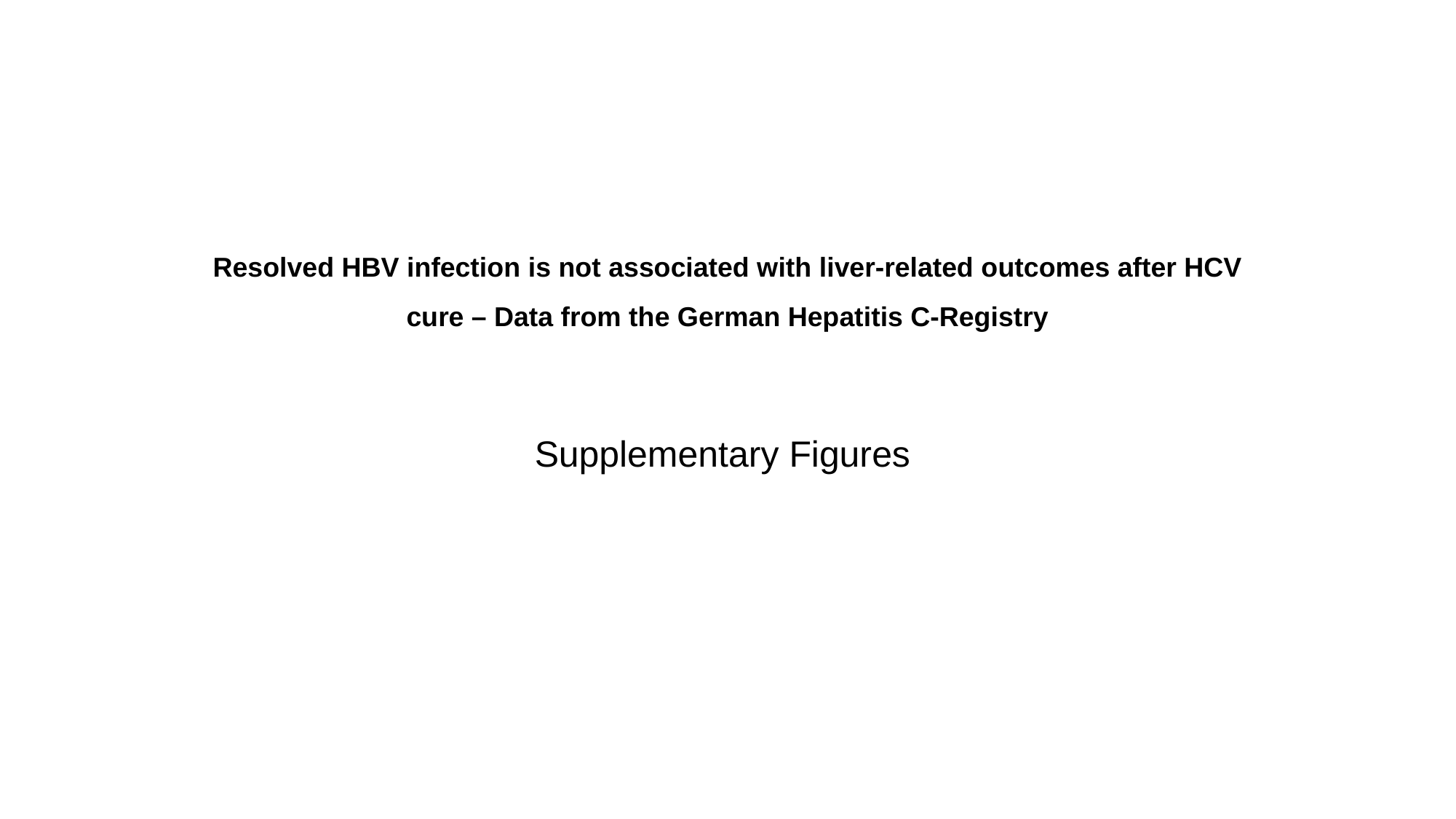

# Resolved HBV infection is not associated with liver-related outcomes after HCV cure – Data from the German Hepatitis C-Registry
Supplementary Figures

## Slide 2
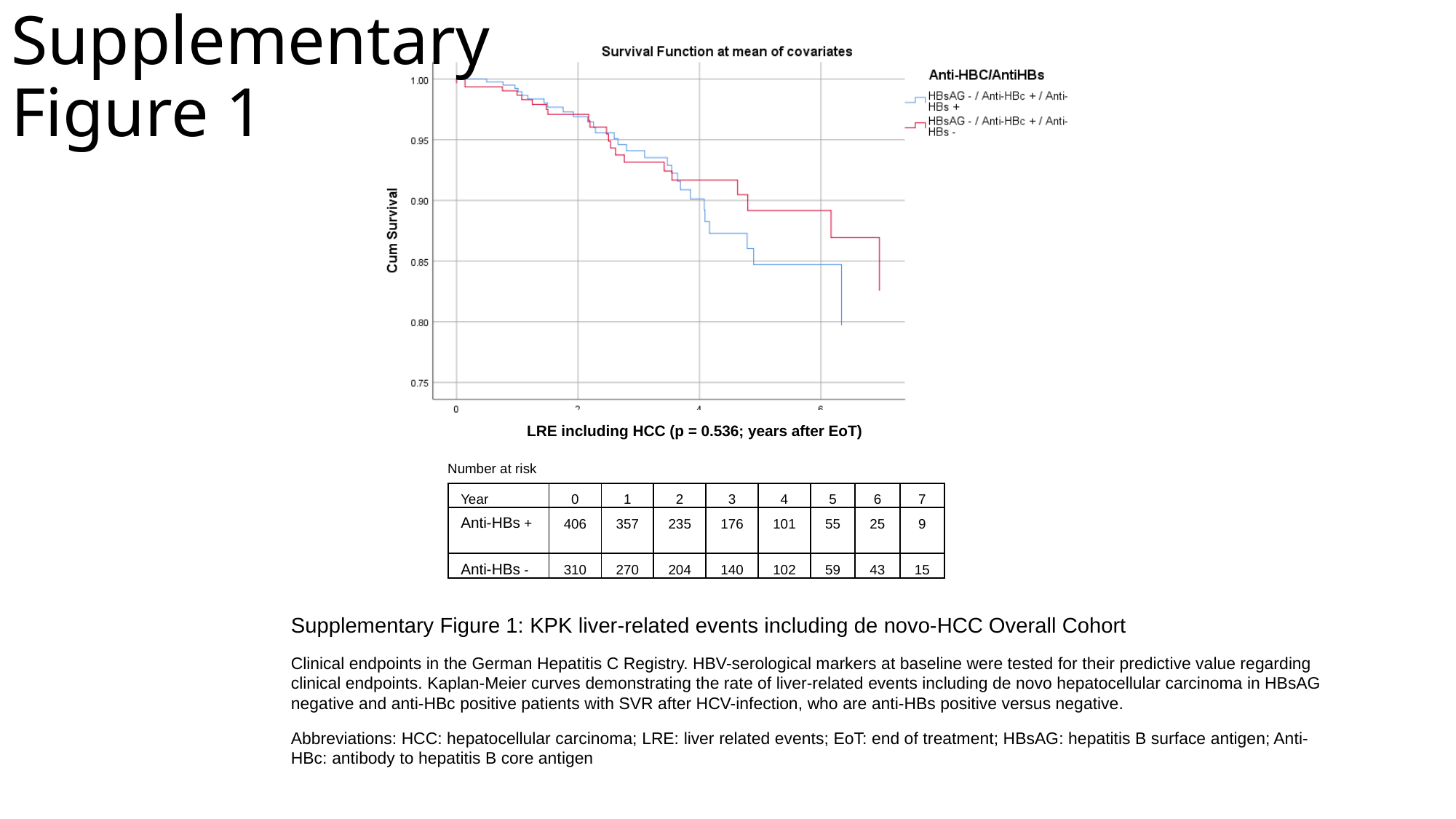

# Supplementary Figure 1
LRE including HCC (p = 0.536; years after EoT)
Number at risk
| Year | 0 | 1 | 2 | 3 | 4 | 5 | 6 | 7 |
| --- | --- | --- | --- | --- | --- | --- | --- | --- |
| Anti-HBs + | 406 | 357 | 235 | 176 | 101 | 55 | 25 | 9 |
| Anti-HBs - | 310 | 270 | 204 | 140 | 102 | 59 | 43 | 15 |
Supplementary Figure 1: KPK liver-related events including de novo-HCC Overall Cohort
Clinical endpoints in the German Hepatitis C Registry. HBV-serological markers at baseline were tested for their predictive value regarding clinical endpoints. Kaplan-Meier curves demonstrating the rate of liver-related events including de novo hepatocellular carcinoma in HBsAG negative and anti-HBc positive patients with SVR after HCV-infection, who are anti-HBs positive versus negative.
Abbreviations: HCC: hepatocellular carcinoma; LRE: liver related events; EoT: end of treatment; HBsAG: hepatitis B surface antigen; Anti-HBc: antibody to hepatitis B core antigen

## Slide 3
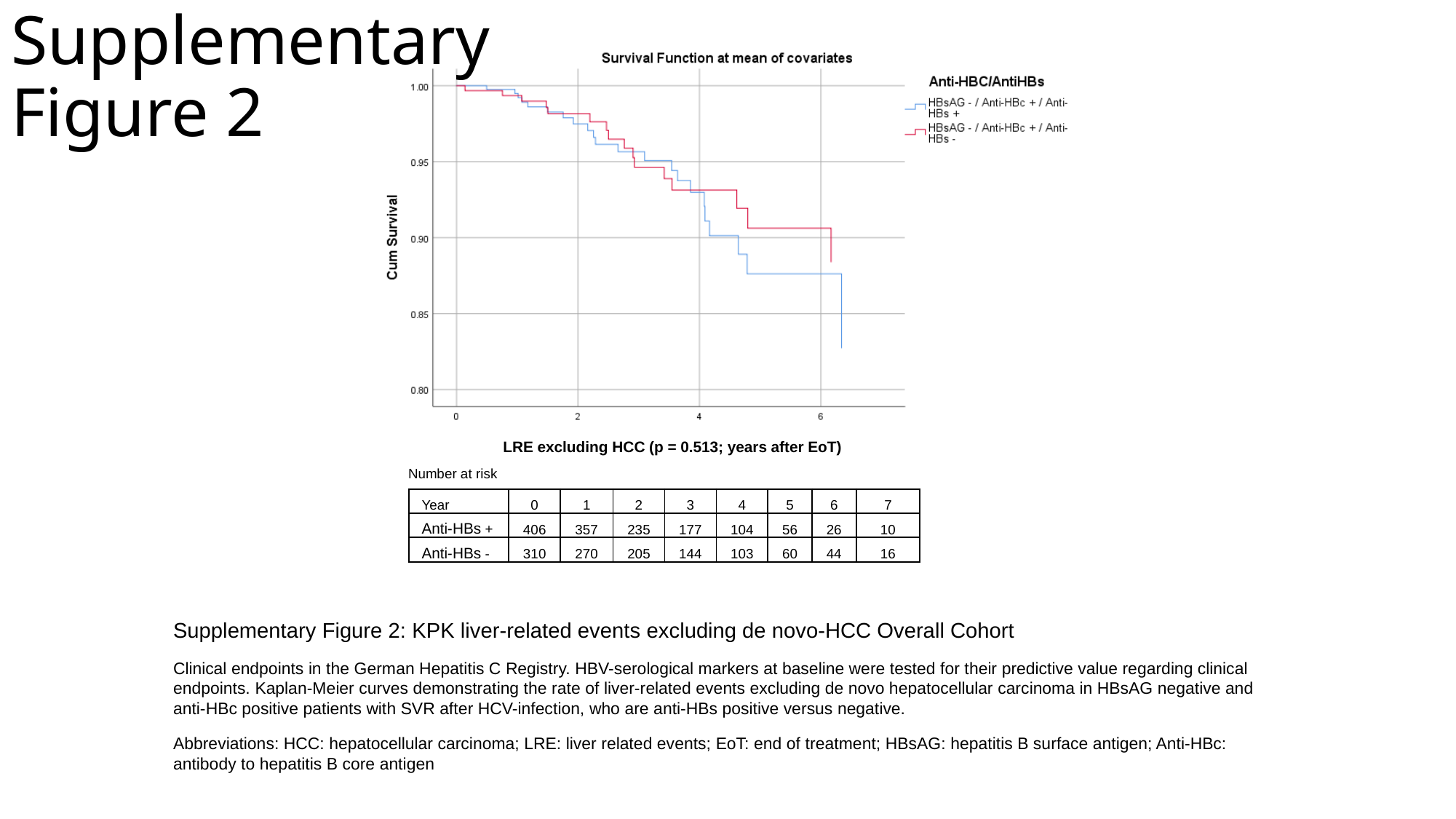

# Supplementary Figure 2
LRE excluding HCC (p = 0.513; years after EoT)
Number at risk
| Year | 0 | 1 | 2 | 3 | 4 | 5 | 6 | 7 |
| --- | --- | --- | --- | --- | --- | --- | --- | --- |
| Anti-HBs + | 406 | 357 | 235 | 177 | 104 | 56 | 26 | 10 |
| Anti-HBs - | 310 | 270 | 205 | 144 | 103 | 60 | 44 | 16 |
Supplementary Figure 2: KPK liver-related events excluding de novo-HCC Overall Cohort
Clinical endpoints in the German Hepatitis C Registry. HBV-serological markers at baseline were tested for their predictive value regarding clinical endpoints. Kaplan-Meier curves demonstrating the rate of liver-related events excluding de novo hepatocellular carcinoma in HBsAG negative and anti-HBc positive patients with SVR after HCV-infection, who are anti-HBs positive versus negative.
Abbreviations: HCC: hepatocellular carcinoma; LRE: liver related events; EoT: end of treatment; HBsAG: hepatitis B surface antigen; Anti-HBc: antibody to hepatitis B core antigen

## Slide 4
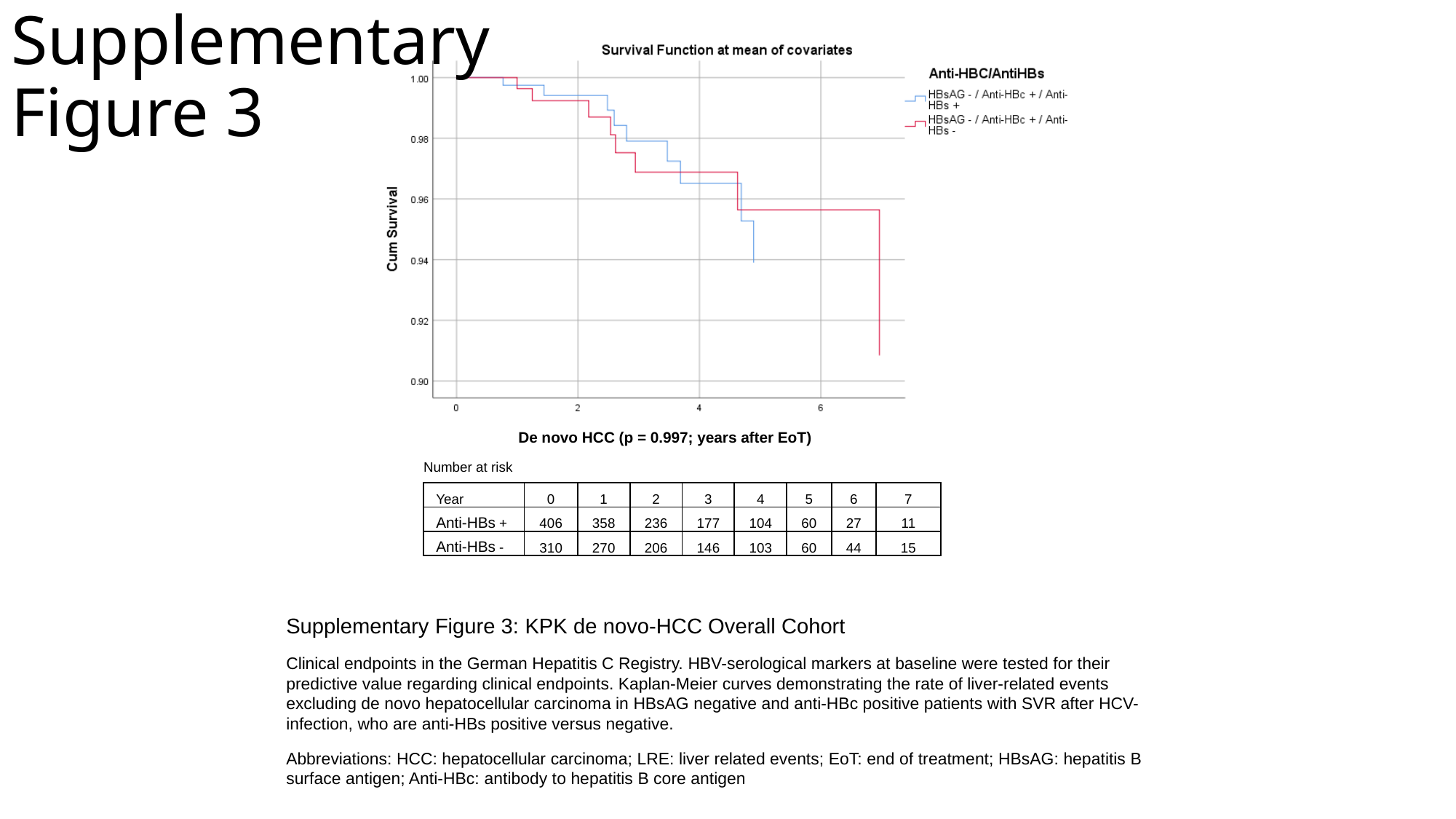

# Supplementary Figure 3
De novo HCC (p = 0.997; years after EoT)
Number at risk
| Year | 0 | 1 | 2 | 3 | 4 | 5 | 6 | 7 |
| --- | --- | --- | --- | --- | --- | --- | --- | --- |
| Anti-HBs + | 406 | 358 | 236 | 177 | 104 | 60 | 27 | 11 |
| Anti-HBs - | 310 | 270 | 206 | 146 | 103 | 60 | 44 | 15 |
Supplementary Figure 3: KPK de novo-HCC Overall Cohort
Clinical endpoints in the German Hepatitis C Registry. HBV-serological markers at baseline were tested for their predictive value regarding clinical endpoints. Kaplan-Meier curves demonstrating the rate of liver-related events excluding de novo hepatocellular carcinoma in HBsAG negative and anti-HBc positive patients with SVR after HCV-infection, who are anti-HBs positive versus negative.
Abbreviations: HCC: hepatocellular carcinoma; LRE: liver related events; EoT: end of treatment; HBsAG: hepatitis B surface antigen; Anti-HBc: antibody to hepatitis B core antigen

## Slide 5
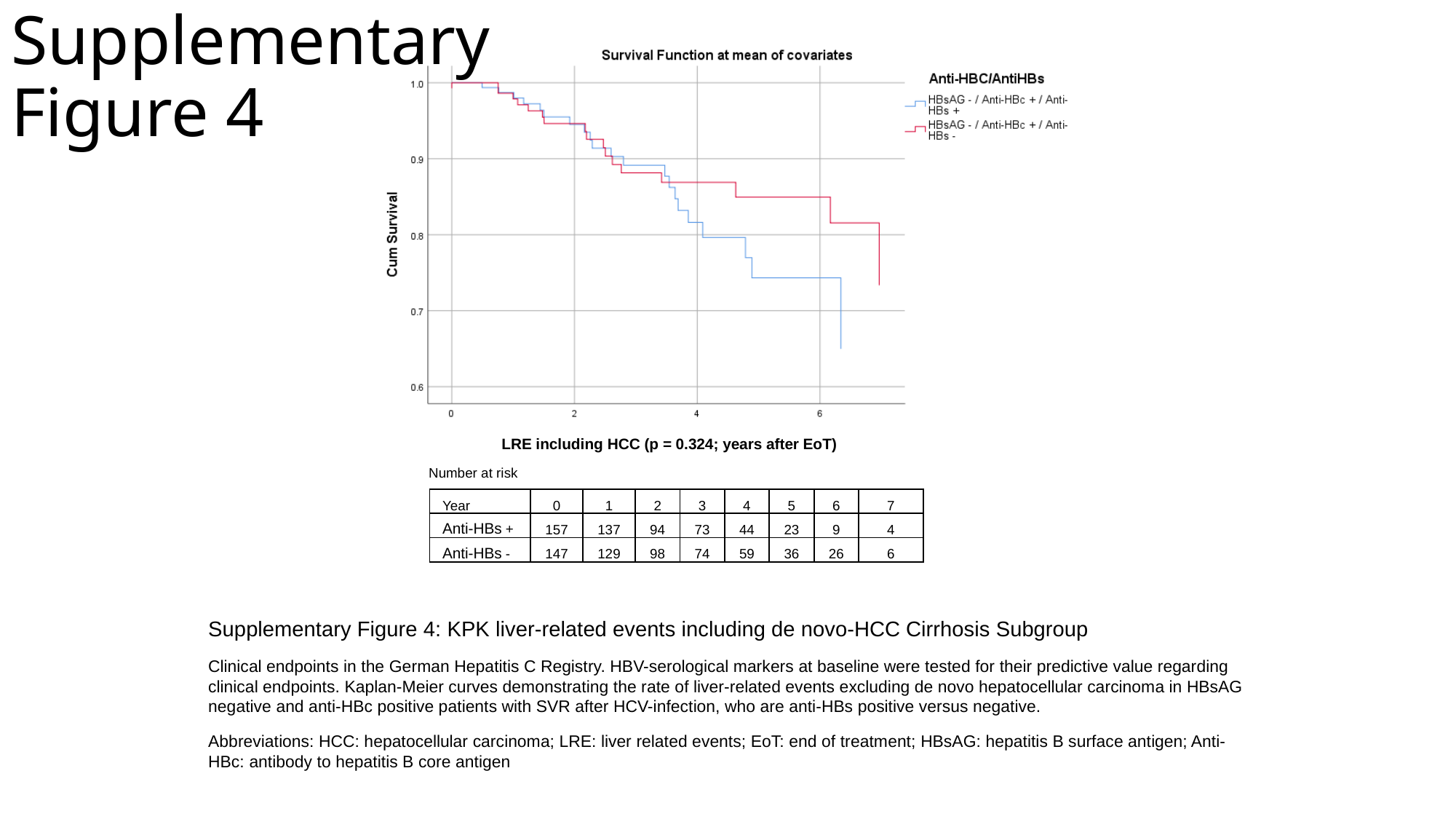

# Supplementary Figure 4
LRE including HCC (p = 0.324; years after EoT)
Number at risk
| Year | 0 | 1 | 2 | 3 | 4 | 5 | 6 | 7 |
| --- | --- | --- | --- | --- | --- | --- | --- | --- |
| Anti-HBs + | 157 | 137 | 94 | 73 | 44 | 23 | 9 | 4 |
| Anti-HBs - | 147 | 129 | 98 | 74 | 59 | 36 | 26 | 6 |
Supplementary Figure 4: KPK liver-related events including de novo-HCC Cirrhosis Subgroup
Clinical endpoints in the German Hepatitis C Registry. HBV-serological markers at baseline were tested for their predictive value regarding clinical endpoints. Kaplan-Meier curves demonstrating the rate of liver-related events excluding de novo hepatocellular carcinoma in HBsAG negative and anti-HBc positive patients with SVR after HCV-infection, who are anti-HBs positive versus negative.
Abbreviations: HCC: hepatocellular carcinoma; LRE: liver related events; EoT: end of treatment; HBsAG: hepatitis B surface antigen; Anti-HBc: antibody to hepatitis B core antigen

## Slide 6
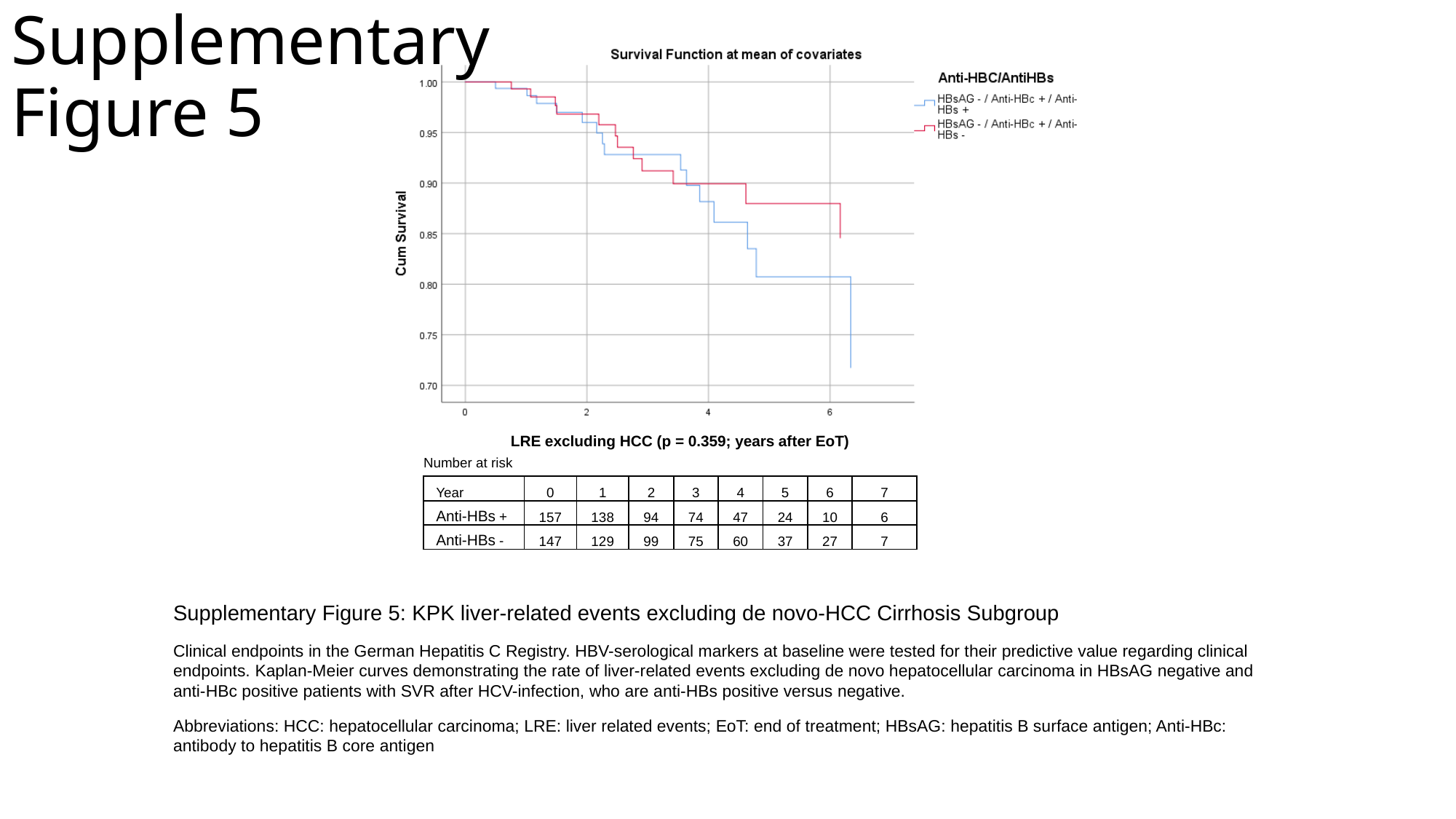

# Supplementary Figure 5
LRE excluding HCC (p = 0.359; years after EoT)
Number at risk
| Year | 0 | 1 | 2 | 3 | 4 | 5 | 6 | 7 |
| --- | --- | --- | --- | --- | --- | --- | --- | --- |
| Anti-HBs + | 157 | 138 | 94 | 74 | 47 | 24 | 10 | 6 |
| Anti-HBs - | 147 | 129 | 99 | 75 | 60 | 37 | 27 | 7 |
Supplementary Figure 5: KPK liver-related events excluding de novo-HCC Cirrhosis Subgroup
Clinical endpoints in the German Hepatitis C Registry. HBV-serological markers at baseline were tested for their predictive value regarding clinical endpoints. Kaplan-Meier curves demonstrating the rate of liver-related events excluding de novo hepatocellular carcinoma in HBsAG negative and anti-HBc positive patients with SVR after HCV-infection, who are anti-HBs positive versus negative.
Abbreviations: HCC: hepatocellular carcinoma; LRE: liver related events; EoT: end of treatment; HBsAG: hepatitis B surface antigen; Anti-HBc: antibody to hepatitis B core antigen

## Slide 7
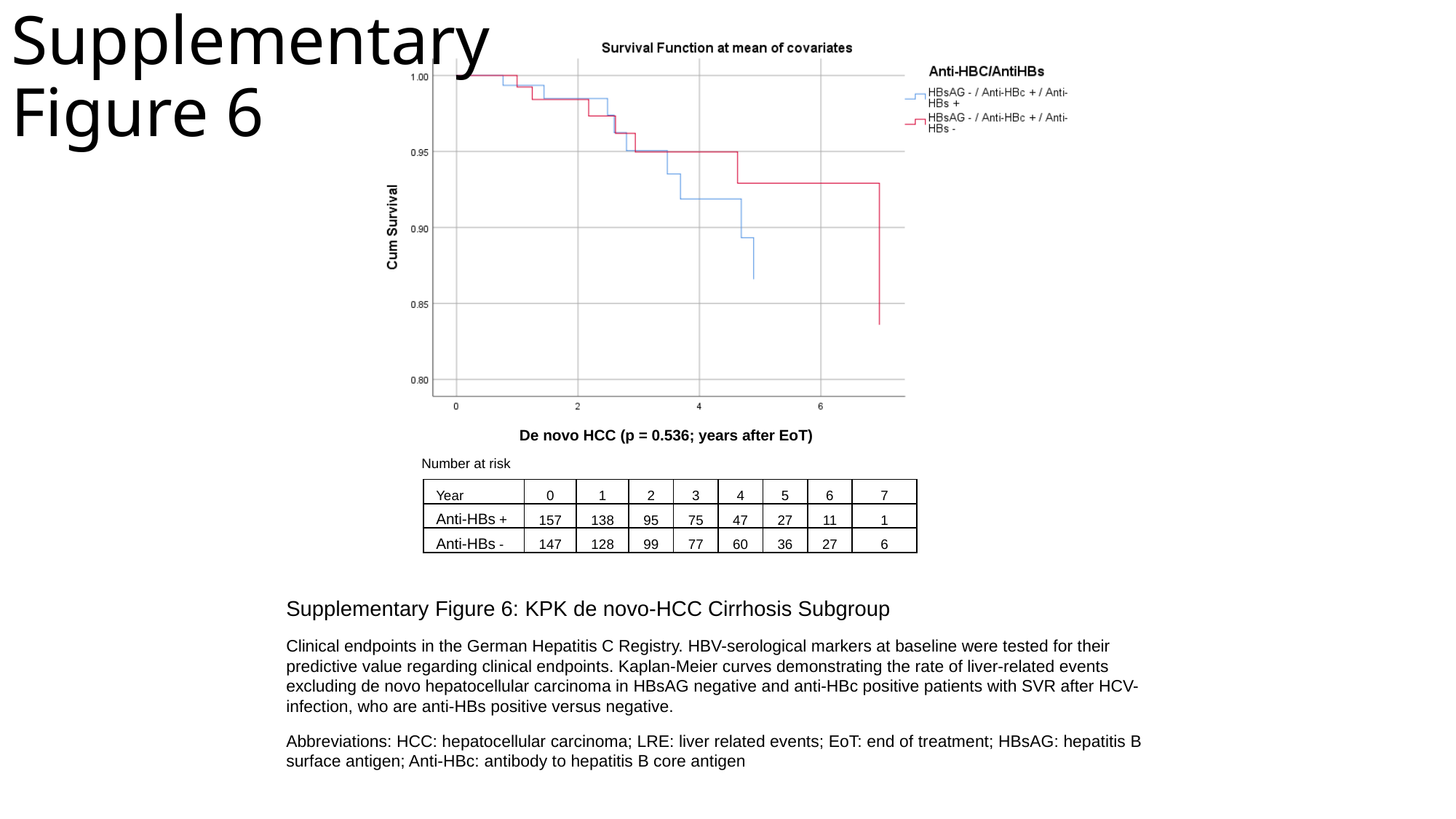

# Supplementary Figure 6
De novo HCC (p = 0.536; years after EoT)
Number at risk
| Year | 0 | 1 | 2 | 3 | 4 | 5 | 6 | 7 |
| --- | --- | --- | --- | --- | --- | --- | --- | --- |
| Anti-HBs + | 157 | 138 | 95 | 75 | 47 | 27 | 11 | 1 |
| Anti-HBs - | 147 | 128 | 99 | 77 | 60 | 36 | 27 | 6 |
Supplementary Figure 6: KPK de novo-HCC Cirrhosis Subgroup
Clinical endpoints in the German Hepatitis C Registry. HBV-serological markers at baseline were tested for their predictive value regarding clinical endpoints. Kaplan-Meier curves demonstrating the rate of liver-related events excluding de novo hepatocellular carcinoma in HBsAG negative and anti-HBc positive patients with SVR after HCV-infection, who are anti-HBs positive versus negative.
Abbreviations: HCC: hepatocellular carcinoma; LRE: liver related events; EoT: end of treatment; HBsAG: hepatitis B surface antigen; Anti-HBc: antibody to hepatitis B core antigen
