# Supplementary material for: Resolved HBV Infection Is Not Associated With Liver‐Related Outcomes in Survival Analysis of Caucasians After HCV Cure
Source: Liver Int. 2026 Apr 30;46:e70620. doi: 10.1111/liv.70620 (PMC13130161; doi:10.1111/liv.70620)
Supplement: Supplementary file 2 — Table S1: Liver‐related events including de novo HCC– Univariate Analysis Overall Cohort. Table S2: Liver‐related events excluding de novo HCC—Univariate Analysis Overall Cohort. Table S3: De novo HCC—Univariate Analysis Overall Cohort. Table S4: Liver‐related events including de novo HCC—Univariate Analysis Cirrhosis Subgroup. Table S5: Liver‐related events excluding de novo HCC—Univariate Analysis Cirrhosis Subgroup. Table S6: De novo HCC—Univariate Analysis Cirrhosis Subgroup. [file LIV-46-0-s001.pptx]

## Slide 1
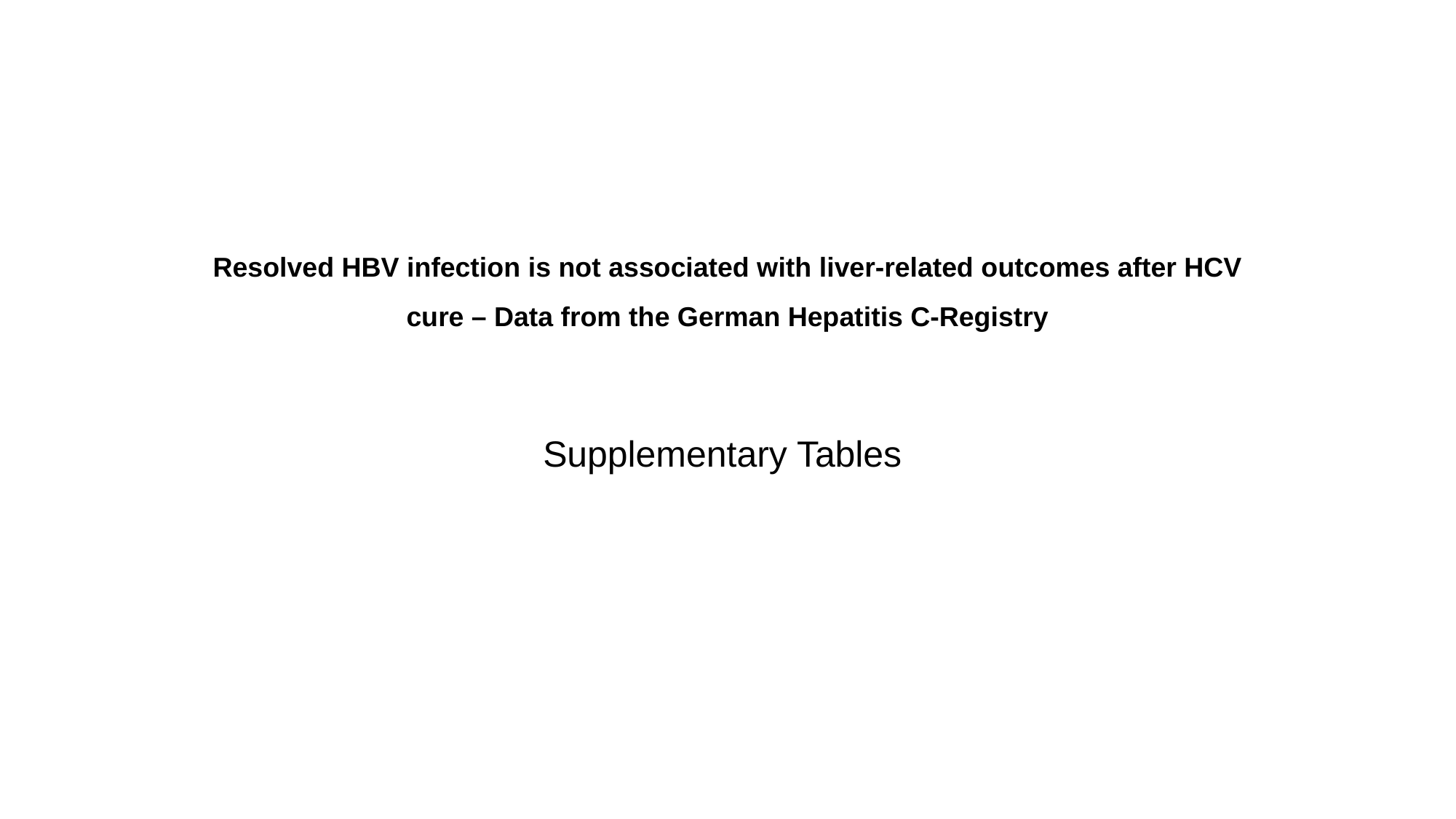

# Resolved HBV infection is not associated with liver-related outcomes after HCV cure – Data from the German Hepatitis C-Registry
Supplementary Tables

## Slide 2
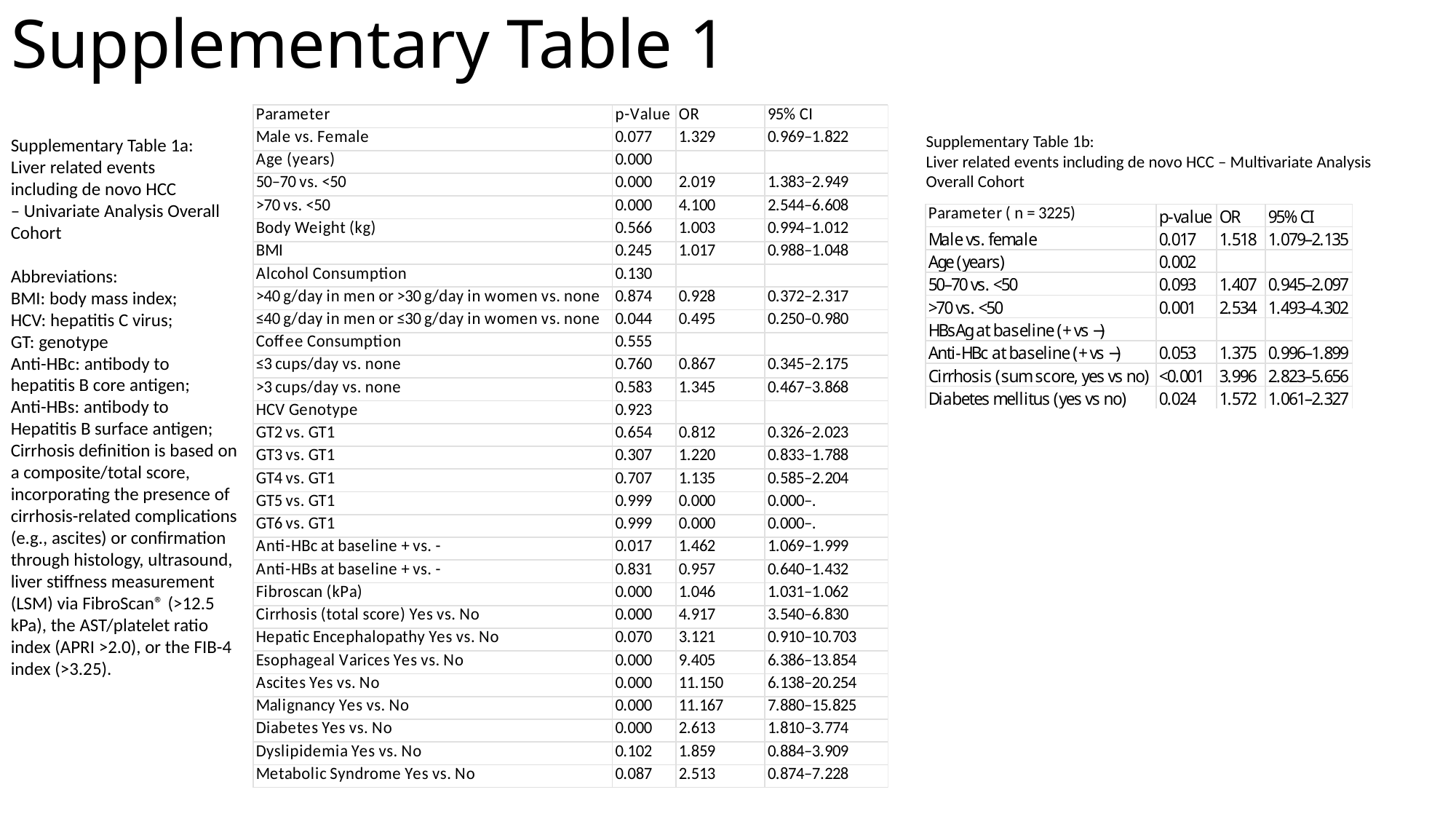

# Supplementary Table 1
Supplementary Table 1b:
Liver related events including de novo HCC – Multivariate Analysis Overall Cohort
Supplementary Table 1a:
Liver related events
including de novo HCC
– Univariate Analysis Overall Cohort
Abbreviations:
BMI: body mass index;
HCV: hepatitis C virus;
GT: genotype
Anti-HBc: antibody to
hepatitis B core antigen;
Anti-HBs: antibody to
Hepatitis B surface antigen; Cirrhosis definition is based on a composite/total score, incorporating the presence of cirrhosis-related complications (e.g., ascites) or confirmation through histology, ultrasound, liver stiffness measurement (LSM) via FibroScan® (>12.5 kPa), the AST/platelet ratio index (APRI >2.0), or the FIB-4 index (>3.25).

## Slide 3
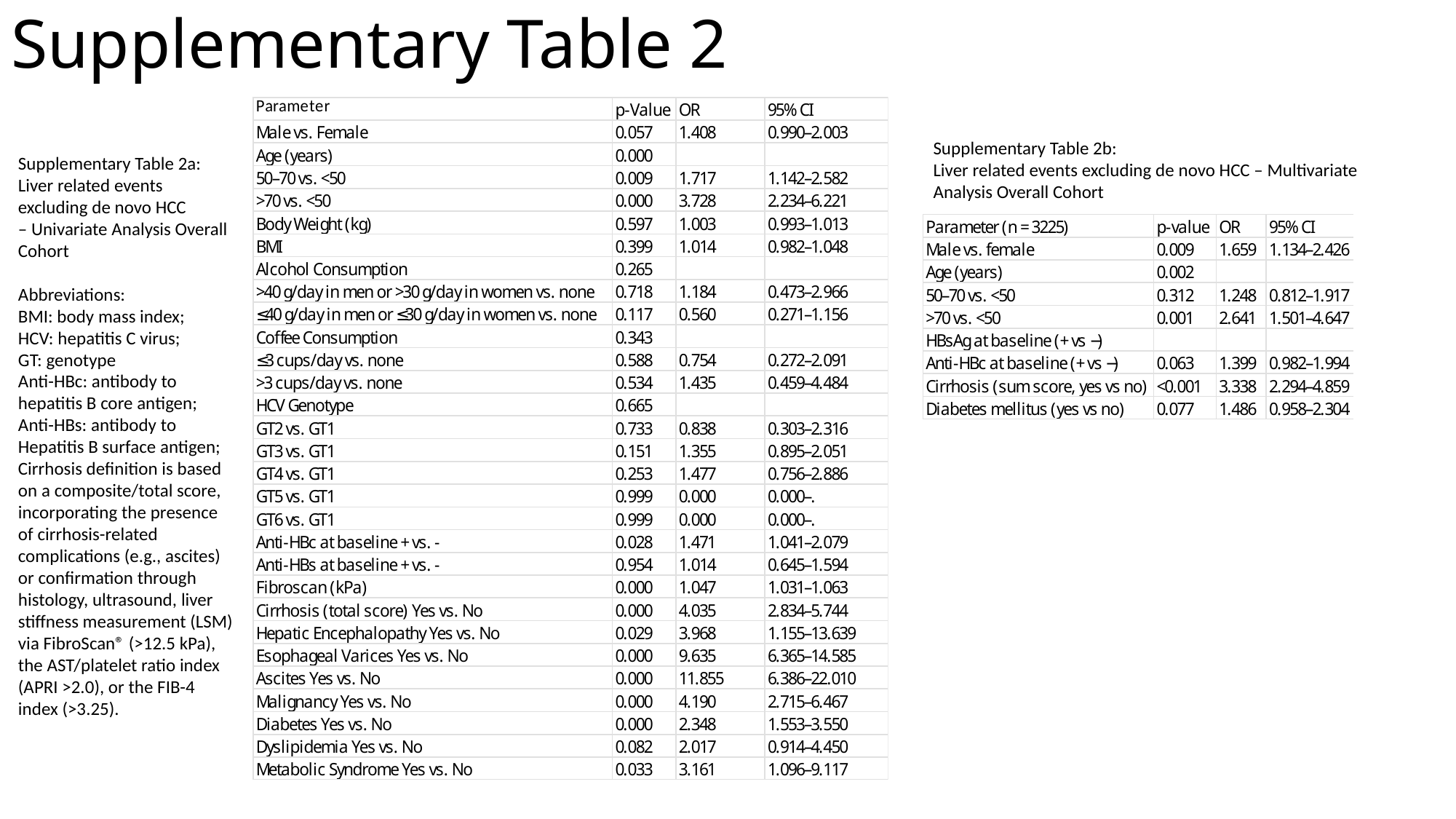

# Supplementary Table 2
Supplementary Table 2b:
Liver related events excluding de novo HCC – Multivariate Analysis Overall Cohort
Supplementary Table 2a:
Liver related events
excluding de novo HCC
– Univariate Analysis Overall Cohort
Abbreviations:
BMI: body mass index;
HCV: hepatitis C virus;
GT: genotype
Anti-HBc: antibody to
hepatitis B core antigen;
Anti-HBs: antibody to
Hepatitis B surface antigen; Cirrhosis definition is based on a composite/total score, incorporating the presence of cirrhosis-related complications (e.g., ascites) or confirmation through histology, ultrasound, liver stiffness measurement (LSM) via FibroScan® (>12.5 kPa), the AST/platelet ratio index (APRI >2.0), or the FIB-4 index (>3.25).

## Slide 4
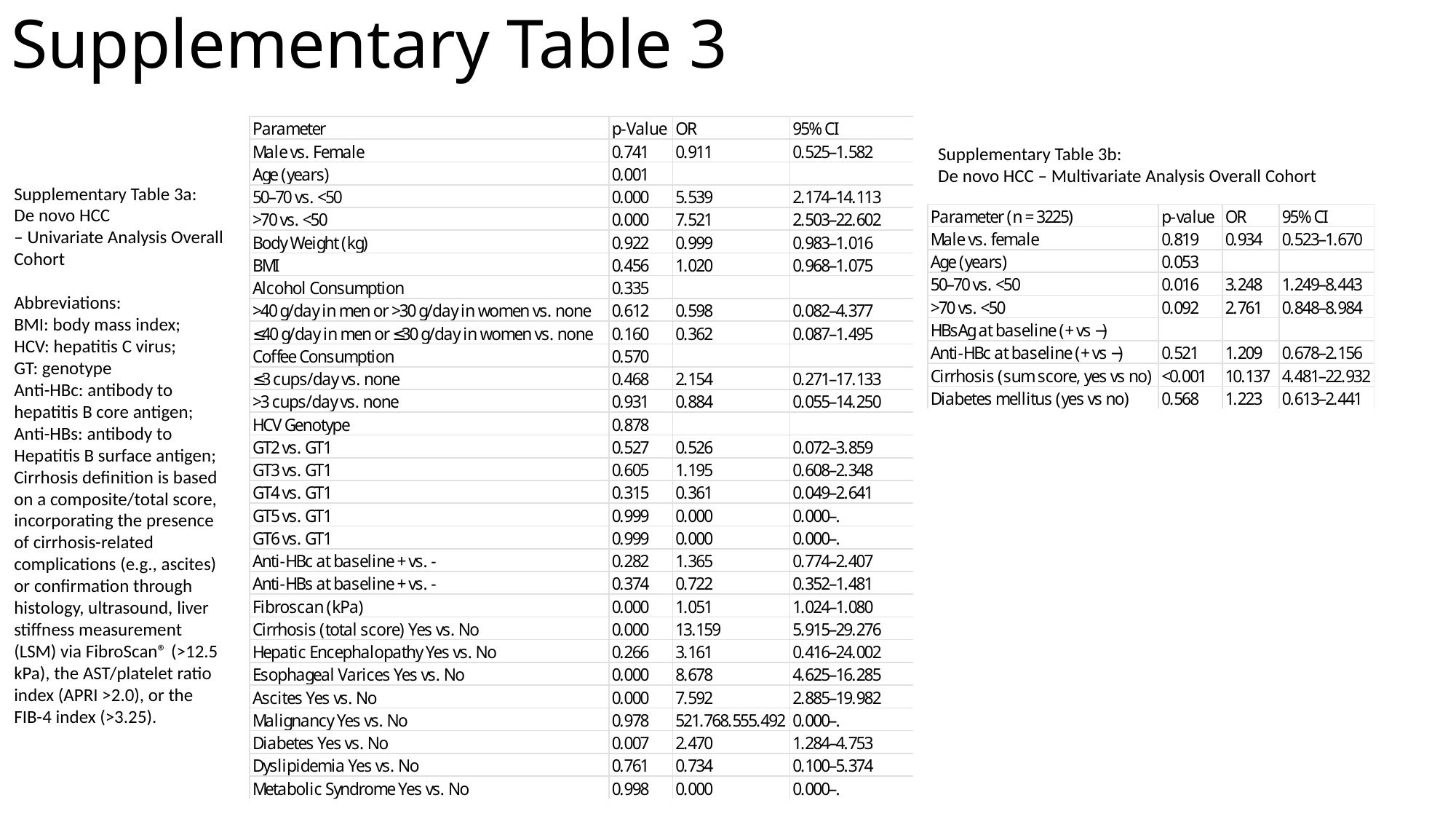

# Supplementary Table 3
Supplementary Table 3b:
De novo HCC – Multivariate Analysis Overall Cohort
Supplementary Table 3a:
De novo HCC
– Univariate Analysis Overall Cohort
Abbreviations:
BMI: body mass index;
HCV: hepatitis C virus;
GT: genotype
Anti-HBc: antibody to
hepatitis B core antigen;
Anti-HBs: antibody to
Hepatitis B surface antigen; Cirrhosis definition is based on a composite/total score, incorporating the presence of cirrhosis-related complications (e.g., ascites) or confirmation through histology, ultrasound, liver stiffness measurement (LSM) via FibroScan® (>12.5 kPa), the AST/platelet ratio index (APRI >2.0), or the FIB-4 index (>3.25).

## Slide 5
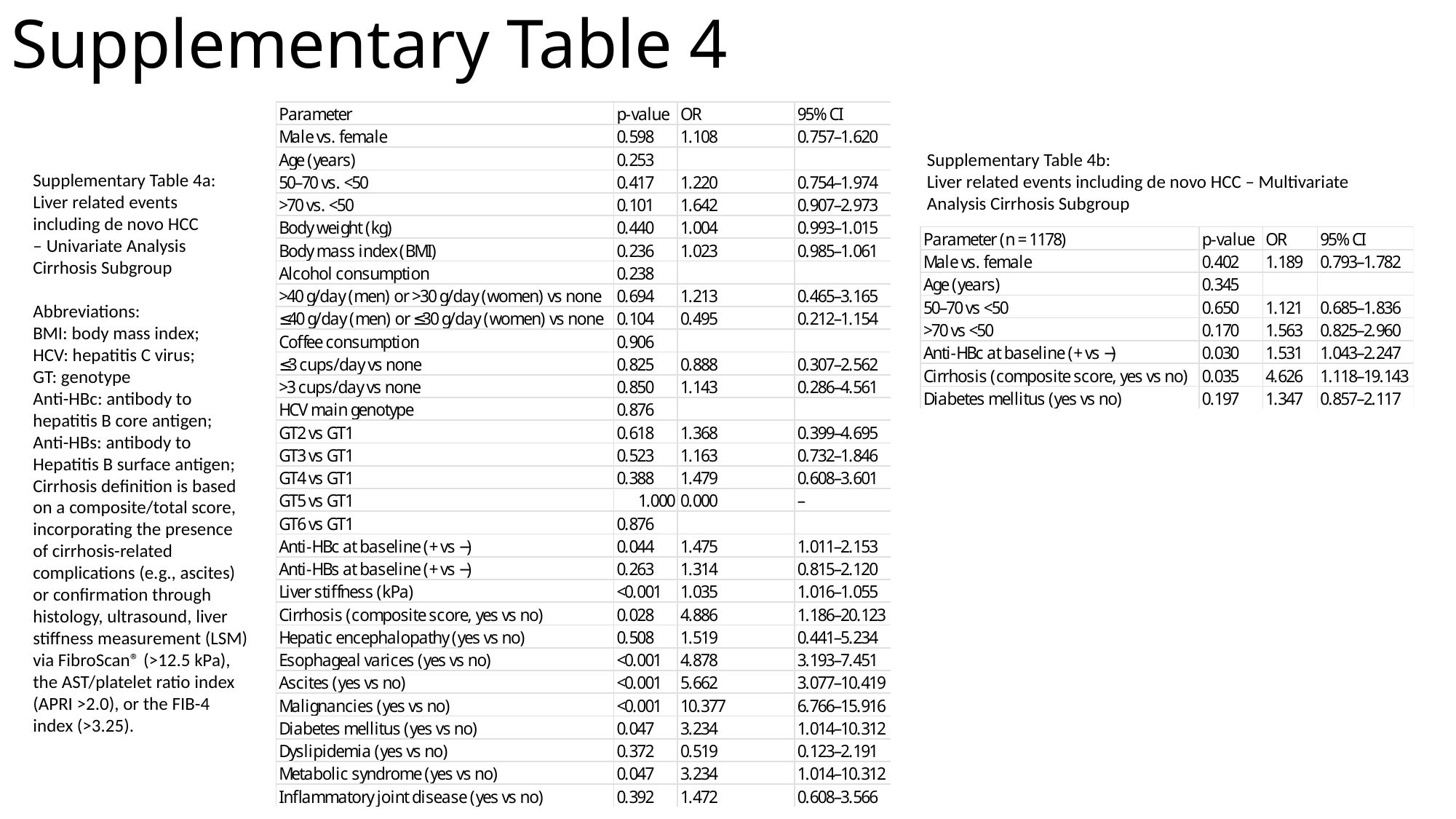

# Supplementary Table 4
Supplementary Table 4b:
Liver related events including de novo HCC – Multivariate Analysis Cirrhosis Subgroup
Supplementary Table 4a:
Liver related events
including de novo HCC
– Univariate Analysis Cirrhosis Subgroup
Abbreviations:
BMI: body mass index;
HCV: hepatitis C virus;
GT: genotype
Anti-HBc: antibody to
hepatitis B core antigen;
Anti-HBs: antibody to
Hepatitis B surface antigen; Cirrhosis definition is based on a composite/total score, incorporating the presence of cirrhosis-related complications (e.g., ascites) or confirmation through histology, ultrasound, liver stiffness measurement (LSM) via FibroScan® (>12.5 kPa), the AST/platelet ratio index (APRI >2.0), or the FIB-4 index (>3.25).

## Slide 6
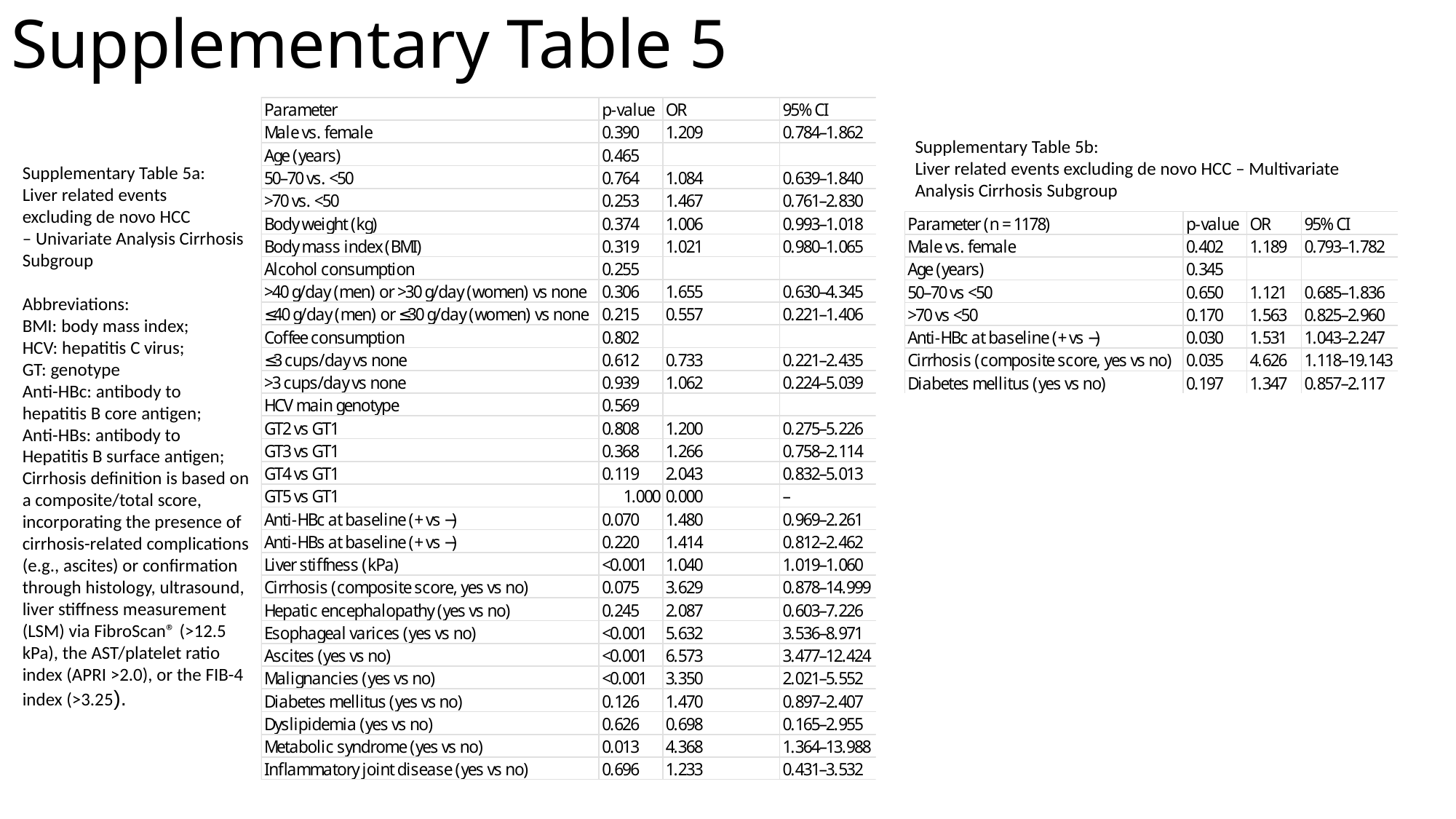

# Supplementary Table 5
Supplementary Table 5b:
Liver related events excluding de novo HCC – Multivariate Analysis Cirrhosis Subgroup
Supplementary Table 5a:
Liver related events
excluding de novo HCC
– Univariate Analysis Cirrhosis Subgroup
Abbreviations:
BMI: body mass index;
HCV: hepatitis C virus;
GT: genotype
Anti-HBc: antibody to
hepatitis B core antigen;
Anti-HBs: antibody to
Hepatitis B surface antigen; Cirrhosis definition is based on a composite/total score, incorporating the presence of cirrhosis-related complications (e.g., ascites) or confirmation through histology, ultrasound, liver stiffness measurement (LSM) via FibroScan® (>12.5 kPa), the AST/platelet ratio index (APRI >2.0), or the FIB-4 index (>3.25).

## Slide 7
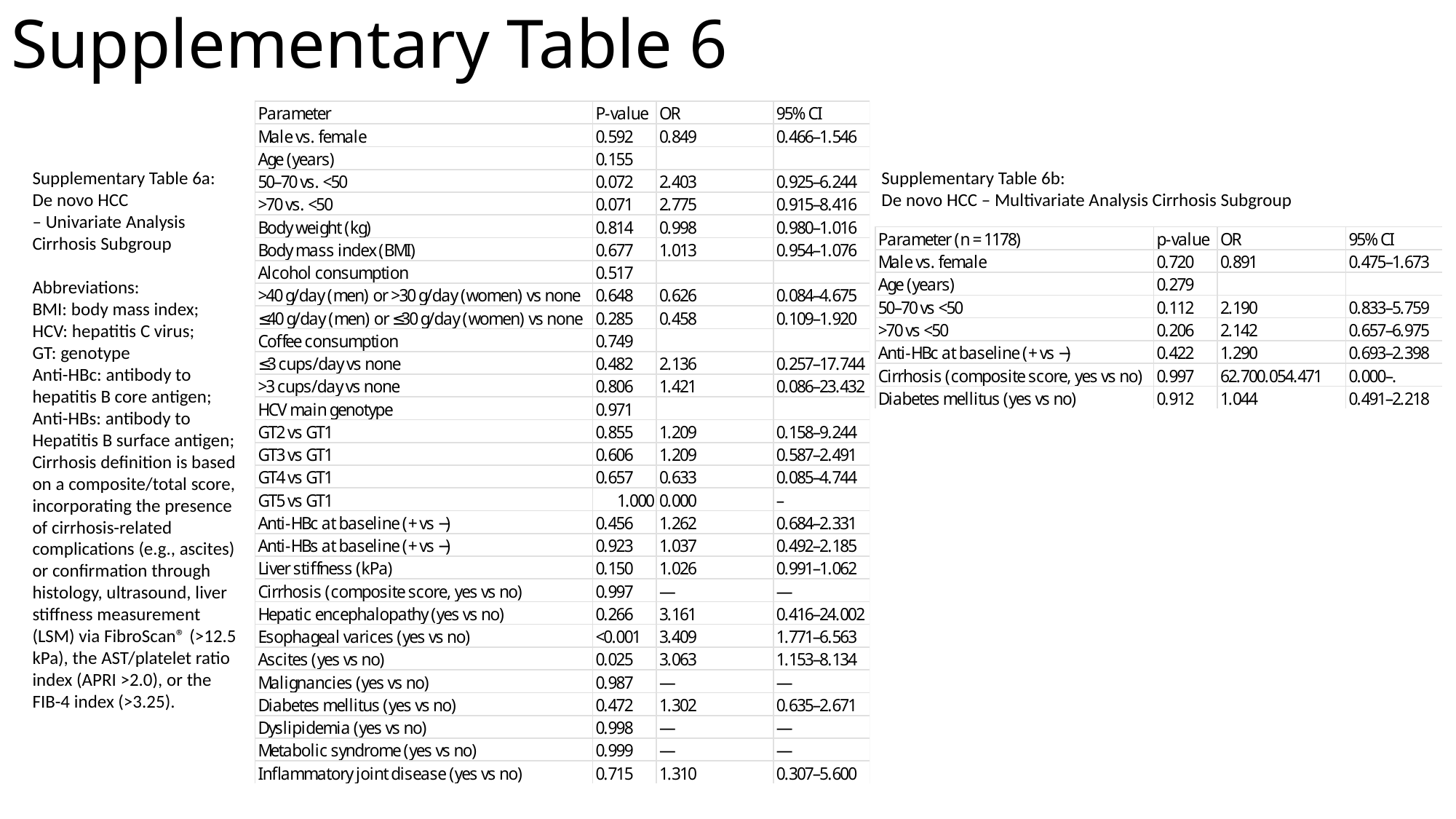

# Supplementary Table 6
Supplementary Table 6a:
De novo HCC
– Univariate Analysis Cirrhosis Subgroup
Abbreviations:
BMI: body mass index;
HCV: hepatitis C virus;
GT: genotype
Anti-HBc: antibody to
hepatitis B core antigen;
Anti-HBs: antibody to
Hepatitis B surface antigen; Cirrhosis definition is based on a composite/total score, incorporating the presence of cirrhosis-related complications (e.g., ascites) or confirmation through histology, ultrasound, liver stiffness measurement (LSM) via FibroScan® (>12.5 kPa), the AST/platelet ratio index (APRI >2.0), or the FIB-4 index (>3.25).
Supplementary Table 6b:
De novo HCC – Multivariate Analysis Cirrhosis Subgroup
